# Single cell and spatial analysis of immune-hot and immune-cold tumours identifies fibroblast subtypes associated with distinct immunological niches and positive immunotherapy response

**DOI:** 10.1186/s12943-024-02191-9

**Published:** 2025-01-06

**Authors:** Benjamin H. Jenkins, Ian Tracy, Maria Fernanda S. D. Rodrigues, Melanie J. L. Smith, Begoña R. Martinez, Mark Edmond, Sangeetha Mahadevan, Anjali Rao, Hailing Zong, Kai Liu, Abhishek Aggarwal, Li Li, Lauri Diehl, Emma V. King, Jamie G. Bates, Christopher J. Hanley, Gareth J. Thomas

**Affiliations:** 1 School of Cancer Sciences, University of Southampton, Southampton, UK; 2 NIHR Experimental Cancer Medicine Centre, University of Southampton, Southampton, UK; 3 Postgraduate Program in Medicine-Biophotonics, Nove de Julho University, São Paulo, Brazil; 4 Dorset Cancer Centre, Poole Hospital NHS Foundation Trust, Poole, UK; 5 Gilead Sciences Inc., Foster City, CA US

## Abstract

Cancer-associated Fibroblasts (CAFs) have emerged as critical regulators of anti-tumour immunity, with both beneficial and detrimental properties that remain poorly characterised. To investigate this, we performed single-cell and spatial transcriptomic analysis, comparing head & neck squamous cell carcinoma (HNSCC) subgroups, which although heterogenous, can be considered broadly immune-hot and immune-cold (human papillomavirus [HPV]+ve and HPV-ve tumours respectively). This identified six fibroblast subpopulations, including two with immunomodulatory gene expression profiles (*IL-11 +* inflammatory [i]CAF and *CCL19 *+ fibroblastic reticular cell [FRC]-like). *IL-11 +* iCAF were spatially associated with inflammatory monocytes and regulated in vitro through synergistic activation of canonical NF-κB signalling by IL-1β and TNF-α. FRC-like were enriched in immune-hot HPV+ve tumours, associated with CD4 + T-cells and B-cells in tertiary lymphoid structures and regulated through non-canonical NF-κB signalling via lymphotoxin. Pan-cancer analysis revealed several ‘iCAF’ subgroups present in both normal and cancer tissues; *IL11 +* iCAF were found in cancers from the gastrointestinal (GI) tract and transcriptomically distinct from iCAFs previously described in pancreatic and breast cancers with greater inflammatory properties; FRC-like fibroblasts were present at low frequencies in all tumour types, and were associated with significantly better survival in patients receiving checkpoint immunotherapy. This work clarifies and expands current literature on immunomodulatory CAFs, highlighting links with important immunological niches.

**Supplementary Information:**

The online version contains supplementary material available at 10.1186/s12943-024-02191-9.

## Introduction

Fibroblasts are ubiquitous cells that assume specialised phenotypes and activation states to play a multifaceted role in health and disease [1]. Although historically regarded as structural cells that principally remodel extracellular matrix (ECM) they are now recognised as key immune sentinel cells capable of initiating, maintaining, and suppressing immune responses in response to pathological stimuli [2].

Cancer-associated fibroblasts (CAF) research has mostly focused on cells with a myofibroblast phenotype (myCAF); these are found in most cancer types, have numerous tumour-promoting functions and are akin to myofibroblasts in fibrotic diseases [1, 3]. However, recent single cell studies have identified transcriptomically distinct CAF subtypes, including inflammatory CAF (iCAF), antigen presenting CAF (apCAF) and metabolic CAF (meCAF) [4–7]. These phenotypes form part of a plastic population that can change states in response to local stimuli; Biffi and colleagues elegantly demonstrated this, switching pancreatic stellate cells between myCAF and iCAF states in vitro by manipulating TGF-β and IL-1 signalling respectively [4]. This plasticity emphasises the role of fibroblasts as key early response cells in tissues. Although iCAF have been identified in several cancer types, including pancreatic ductal adenocarcinoma (PDAC), breast and lung cancers [5–7], it is not yet clear whether this phenotype is common to all tumours or whether all ‘iCAF’ are the same. Although there are common mechanisms that fibroblasts use to regulate tissue inflammation, inflammatory stimuli have been shown to produce organ-specific immune signatures in fibroblasts from different organs [8] suggesting that inflammatory CAF phenotypes could vary.

The clinical success of immune checkpoint inhibitors in treating multiple cancer types is well established. However, only a subset of patients respond favourably [9], and this has generated significant interest in understanding how the tumour microenvironment suppresses anti-tumour immunity. myCAF have several immunosuppressive functions and myCAF-rich tumours are resistant to immunotherapy [6, 10, 11]. iCAF also express several cytokines associated with immune evasion, including IL6, LIF and CXCL12 [12]. Conversely, in autoimmunity, fibroblasts have been shown to amplify chronic inflammation [13], and a novel population of ‘interferon licenced fibroblasts’ that enhance immunotherapy response has been identified in murine tumour models [14]. Thus, a fibroblast may support or suppress immunity depending on context. Given their plasticity, the concept of generating an immune-supportive phenotype to improve immunotherapy response in cancer is intriguing.

In most cancer types, tumours with high levels of tumour-infiltrating lymphocytes (TIL) have better prognosis and show improved response to checkpoint immunotherapy [15]. Head and neck cancer (HNSCC) is subdivided into human papillomavirus (HPV)-related (HPV+ve) tumours and those typically associated with smoking/alcohol (~ 30% and 70% of cases respectively). Around 85% of HPV+ve HNSCC are heavily infiltrated by T- and B-cells and are considered immune-hot [16]. Despite presenting mostly at late stage, such tumours are associated with significantly better survival compared with TIL-low HPV-ve tumours [16].

In this study we hypothesised that fibroblast phenotypes vary between immune-hot (HPV+ve) and immune-cold (HPV-ve) HNSCC subtypes, reflecting their multifaceted role in anti-tumour immunity. Using single cell and spatial transcriptomics we identified six fibroblast subsets in HNSCC, including two that were characterised by expression of immunomodulatory genes and occupied distinct immunological niches (*IL11* + inflammatory [i]CAF and *CCL19 +* fibroblastic reticular cell [FRC]-like). *IL11* + iCAF were spatially associated with and activated by inflammatory monocytes, through IL-1β and TNF-α stimulation. We also showed that *IL11* + iCAF were transcriptomically distinct from iCAFs previously described in pancreatic and breast cancers [5, 6, 17, 18], with heightened inflammatory features. FRC-like cells were enriched in immune-hot HPV+ve tumours, associated with CD4 + T-cells and B-cells in tertiary lymphoid structures (TLSs), activated by lymphotoxin signalling. Across cancer types, FRC-like cells represented a rare phenotype, but were detected in all tumour types and associated with positive response to checkpoint immunotherapy.

## Methods

### Human subjects

Ethical approval for the study was obtained through the UK National Research Ethics Service (REC No. 09/H0501/90) and written informed consent was obtained from all subjects. Tumour and matched-normal tissue were obtained from patients undergoing surgical tumour resection at Poole Hospital (Poole, Dorset, UK) for HNSCC. Tissue samples were transported (within 1 h) to the laboratory on ice in serum-free Dulbecco’s Modified Eagle Medium (DMEM; Sigma-Aldrich). Sample information (including clinical and sample digestions) is shown in Supplementary Table 1. Patient HPV status was confirmed using p16 immunostaining in combination with assessing HPV-encoded gene expression using a human-HPV hybrid reference genome to align and map reads (see details below). HPV-encoded exons were detected in 6 patients using the human-HPV-16 reference genome and 1 patient using the human-HPV-33 reference genome (Supplementary Fig. 1A).

### Primary fibroblast culture and in vitro experiments

Please see Supplementary Materials.

### Sample processing

Please see Supplementary Materials.

### scRNA-Seq

For each sample, 5000 single cells were captured on an Illumina 10X Chromium Controller™ system using the Illumina single cell 3’ gene expression and library preparation kits (V3.1 #1000269). Sample capture, sample indexing, and library preparation were carried out according to manufacturer’s instructions. Size distribution, quality control, and quantification of the libraries was assessed using High Sensitivity DNA chips (Agilent Technologies #5067 − 4626) and KAPA library quantification qPCR kit (Roche #07960140001). Prepared libraries were pooled and sent to Oxford Genomics (UK) for 150-base pair, paired-end sequencing on a Novaseq6000™.

### Sequence alignment and annotation

FASTQ files were aligned to the Human reference genome (GRCh38–2020-A) which had the HPV genome concatenated to both the FASTA and GTF reference files (using cellranger count v6.1.1, 10x Genomics). Human-HPV references were made using the cellranger mkref command (cellranger v6.1.1). scRNA-Seq data was processed with cellranger count (cellranger v6.1.1) generating feature-barcode matrices in which subsequent data analysis was carried out in R (v4.1.1) using Seurat package (v4.1.0).

### Quality control, normalisation and integration

Each patient expression matrix was initially created into a Seurat object with cells requiring expression of at least 200 genes and genes expressed in at least 3 cells. Poor quality cells were removed using a mitochondrial RNA percentage threshold calculated by the median + 3* median absolute deviation [cells above this threshold (~ 20%) were removed]. Cells expressing > 6000 features were removed to reduce potential doublets. Seurat was then used for normalisation and reciprocal PCA (RPCA) integration of scRNA-Seq data (further details in Supplementary Materials). Principal component analysis (PCA) was then performed on the integrated object followed by Uniform Manifold Approximation and Projection (UMAP) visualisation. Clustering was performed using shared nearest-neighbour (SNN) graph construction (FindNeighbors) followed by FindClusters.

### Identifying marker genes

Differentially expressed genes (DEGs) were identified for each cluster using FindAllMarkers (Wilcoxon rank sum test) with genes selected expressed in ≥ 25% of cells and log2FC ≥ 0.5 (adjusted p value < 0.05). DEGs were compared to known cell type markers described widely in the literature to annotate broad and finer cell types.

### HNSCC inter-dataset integration

Seurat’s RPCA integration was also used for HNSCC inter-dataset integration with GSE164690 (Further details in Supplementary Materials).

### Gene module scores and pathway analysis

Module scores were calculated using Seurat’s AddModuleScore function calculated by taking the average expression levels of each cluster at the single cell level subtracted by aggregated expression of control feature sets. All gene signatures used in the analysis are shown in Supplementary Table 8. Pathway analysis was performed using over-representation analysis (enrichr v3.2; [19]) and gene set enrichment analysis (GSEA) (clusterprofiler v4.6.2; [20]) using KEGG and MSigDB Hallmark databases. Enriched pathways with P value and adjust p value < 0.05 were examined. PROGENy: Pathway RespOnsive GENes for activity inference was used to infer activities of 14 pathways [21]. PROGENy pathway activity scores were calculated for the Seurat object running ‘progeny’ command (organism="Human”, top = 500, perm = 1, return_assay = TRUE). Pathway activity scores were then scaled. Summarised scores (mean) of each activity for each cell cluster were determined and plotted in a heatmap.

### Differential abundance

We utilised MiloR (v1.6.0) to identify differentially abundant phenotypes using KNN graphs [22]. MiloR was run on the integrated objects separately with the following parameters used for buildGraph and makeNhoods: k = 70, d = 20, refined = TRUE. To account for cell type abundance differences resulting from use of different digestions, the digest was specified in the design formula along with source (tumour/normal), HPV status or lymphocyte abundance classification (see below). SpatialFDR threshold (alpha) was set to 0.05 when highlighting differentially abundant neighbourhoods – which were displayed in bee-swarm plots.

Prior to differential abundance testing of fibroblasts, scRNA-Seq samples were classified into ‘High’, ‘Moderate’ and ‘Low’ tertiles reflective of lymphocyte presence. Pearson residuals for lymphocyte abundance were calculated relative to the total number of immune cells per sample. Samples classified as lymphocyte-High, representing immune-hot tumours were then compared to lymphocyte-Low samples (immune-cold).

When calculating relative cell type proportions per sample in the scRNA-Seq data we accounted for digestion differences in select samples that had undergone scRNA-Seq of liberase and col + digests separately, calculating pseudo-mixtures by combining the relative abundance of cell types for each sample in a 1:9 (liberase: col+) ratio.

### Trajectory and pseudotime

Monocle 3 (v1.0.0) and slingshot (v2.6.0) were used for trajectory analysis and pseudotime calculations. Both methods yielded the same lineages. Monocle 3 was used to determine genes that change as a function of pseudotime, graph_test, specifying the neighbour_graph as ‘prinicpal_graph’ was run.

### Transcription factor analysis

DoRothEA regulons, a collection of transcription factors and their targets, were used to infer transcription factor activities in fibroblast populations [23]. Activities were determined using run_wmean from the decoupleR (v2.5.0) package and subsequently scaled.

### Pan-cancer fibroblast atlas (PCFA) and label transfer

Please see Supplementary Materials.

### Spatial transcriptomics

All pre-sequencing procedures were carried out following the manufacturer’s instructions on 6.5 mm capture areas using the Visium V2 CytAssist workflow. All samples were processed through the Spaceranger pipeline (v2.0.0) according to 10x Genomics guidelines. Please see Supplementary Materials for details of spatial transcriptomics processing, spot deconvolution and spatially guided ligand-receptor/NicheNet analysis.

### Bulk RNA sequencing (scRNA-Seq)

Counts for the Head and Neck Squamous Cell Carcinoma, TCGA-HNSC (https://www.cancer.gov/tcga) (566 samples: 520 primary solid tumour; 46 solid tissue normal) cohort (Illumina HiSeq platform) were downloaded using TCGAbiolinks and converted to CPM using edgeR. TPM normalised data for TCGA-HNSC was downloaded from GDC data portal. The cBioPortal for cancer genomics [24] was used to obtain additional metadata from the Head and Neck Squamous Cell Carcinoma (TCGA, Firehose Legacy) study. UCSCXenaTools package (v1.4.8) was used to download Batch effects normalized mRNA data (*n* = 11,060) from the Pan-Cancer Atlas Hub and corresponding clinical metadata.

### Bulk RNA-Seq deconvolution

To investigate the cell type abundance in bulk RNA-Seq data, the immunedeconv R package (v2.1.0) was used to run MCP-counter [25] on TPM normalised HNSCC (TCGA) Bulk RNA-Seq. The ‘deconvolute’ function was run specifying ‘MCP_counter’.

### ssGSEA

We used single sample GSEA (ssGSEA) using the GSVA package (v1.46.0) to calculate enrichment scores for gene sets in bulk RNA-Seq samples.

### Analysis of immunotherapy data

The following bulk RNA-Seq datasets were used for analysis of immunotherapy treated patients. HNSCC (GSE159067): 102 patients with advanced HNSCC treated with immunotherapy targeting PD-1/PD-L1. Lung (GSE161537): 82 patients with advanced non-small cell lung cancer (NSCLC) treated with second-line immunotherapy targeting PD-1/PD-L1. Metastatic melanoma (PRJEB23709): 91 patients treated with anti-PD-1 alone or combined anti-PD-1 and anti-CTLA-4 immunotherapy. Overall survival analysis (Kaplan-Meier and cox regression) was performed out using survival package (v3.5-7); patients were split into high and low (based on ssGSEA scores) using the optimal cut points determined by surv_cutpoint (survminer v0.4.9). Multivariate cox regression was carried out using ‘coxph’ function (survival) specifying the fibroblast abundance, sex, and age. Generation of fibroblast subset specific gene signatures (Supplementary Table 8) used in ssGSEA are detailed in Supplementary Materials.

### Multiplex immunofluorescence using PhenoCycler-Fusion

Please see Supplementary Materials.

### Statistical analysis

Statistical analysis was performed using R environment v4.1.1 [ggpubr package (v0.4.0) for plotting graphs] and Graph Pad Prism 9 (v10, GraphPad, San Diego, CA, USA). Wilcoxon rank-sum test (two-sided) or Students t-test (two-sided) were used to evaluate associations between continuous variables. Normality was assessed by Shapiro–Wilk test. One-way ANOVA or Kruskal-Wallis test was used to compare > 2 groups. Multiple comparisons were investigated by adjusting the p-value using the Bonferroni method. Correlation analysis was carried out using spearman’s rho (two-sided). P-values were combined using the weighted Fisher’s method. Survival analysis was carried out using Kaplan-Meier curves with log-rank test and multivariate cox regression statistics. *P* < 0.05 was considered to indicate a statistically significant difference.

## Results

### HPV+ve HNSCC frequently has an immune-hot tumour microenvironment

**Fig. 1:**
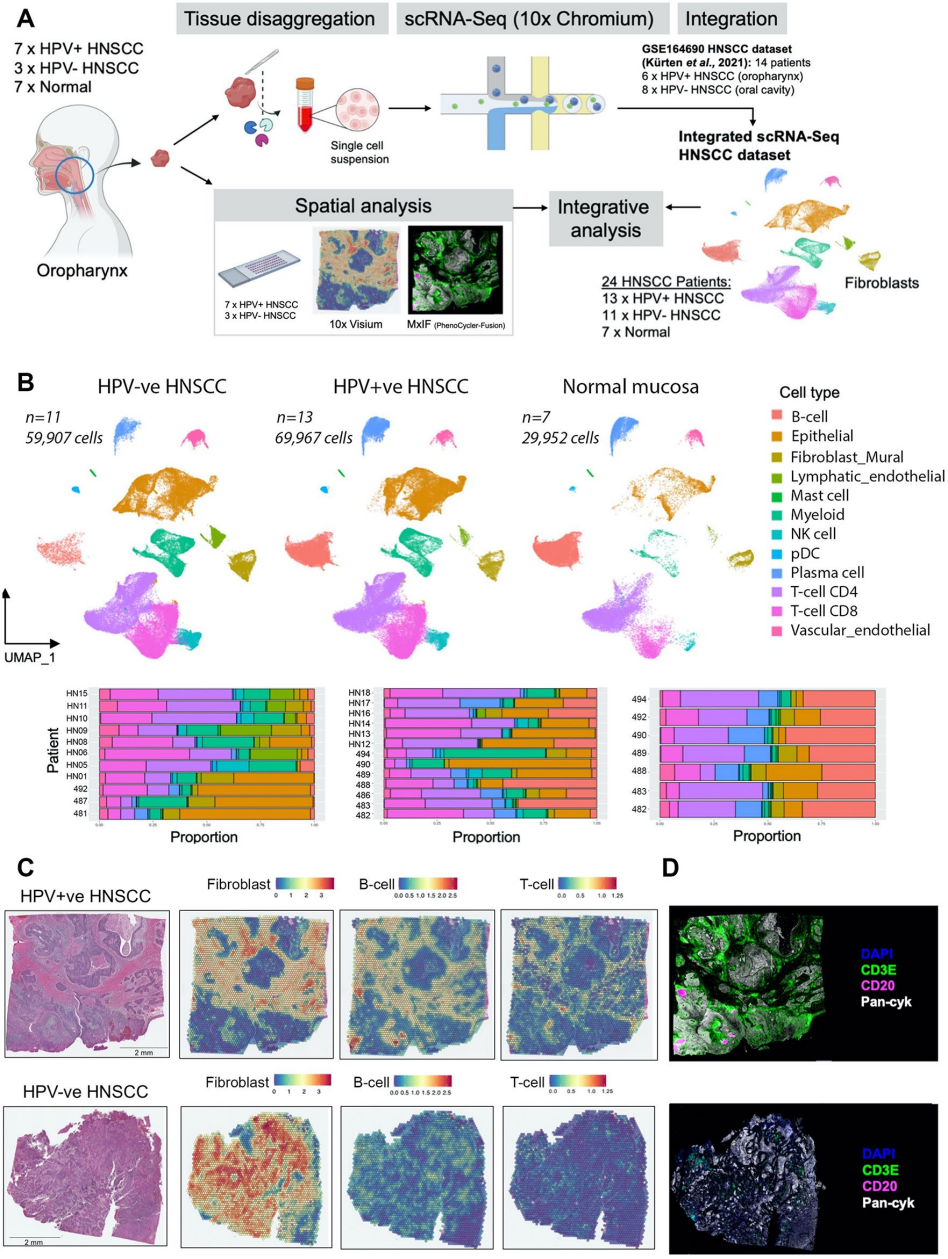
HPV+ve HNSCC frequently has an immune-hot tumour microenvironment. (**A**) Schematic of workflow for integrative single cell and spatial analysis. (**B**) Plot showing UMAP embeddings for integrated (Seurat RPCA) HNSCC scRNA-Seq dataset comprising HPV-ve HNSCC (*n* = 11; 59,907 cells), HPV+ve HNSCC (*n* = 13; 69,967 cells) and normal oropharyngeal tissue (*n* = 7; 29,952 cells). UMAP plots displaying 12 clusters are accompanied with bar plots showing relative proportions of broad cell types per patient sample. Clusters are annotated based on expression of marker genes as shown in Supplementary Fig. 1E. (**C**) H&E images with spatial feature plots showing spatial transcriptomics MCP-counter deconvoluted abundance for B-cells, T-cells and CAF in representative examples of HPV+ve and HPV-ve patients. Cell type abundance within each Visium (10x) spot estimated by MCP-counter is displayed. (**D**) MxIF (Phenocycler-Fusion) examples of DAPI, CD3E, CD20 and Pan-Cytokeratin staining in representative HPV+ve and HPV-ve patients

We set out to characterise fibroblast phenotypes across immune-hot and immune-cold tumours. Previous studies have reported increased lymphocyte infiltration into HPV+ve HNSCC compared to HPV-ve [16]. In order to capture this heterogeneity in immunological contexts, we performed scRNA-Seq on treatment-naïve HNSCC samples (*n* = 10; 7 HPV+ve; 3 HPV-ve) with matched normal oropharyngeal mucosa [*n* = 7] (Fig. 1A; Supplementary Fig. 1B, C; Supplementary Table 1). To increase patient numbers, this (EPG) dataset (82,844 cells after quality control) was integrated with a publicly available dataset (Fig. 1A; HNSCC samples of oral cavity/oropharynx; [26]), generating an atlas of 159,826 cells from 24 patients (11 HPV-ve; 13 HPV+ve; 7 normal; Fig. 1B; Supplementary Fig. 1D, E). Spatial transcriptomic analysis (10x; Visium) and multiplexed immunofluorescence (MxIF) staining and imaging (Akoya; PhenoCycler) were performed on tumour sections from the initial ten patients (Fig. 1A).

Deconvolution of bulk RNA-Seq data from the HNSCC TCGA cohort using MCP-counter confirmed that HPV+ve tumours contain significantly more CD8 + T-cells (*p* < 0.0001), CD4 + T-cells (*p* < 0.0001) and B-cells (*p* < 0.0001; Supplementary Fig. 1F). Spatial transcriptomic analysis using MCP-counter to deconvolute cell type abundance within individual spots, also showed significantly more T-cells (*p* < 0.001), B-cells (*p* < 0.01) and total lymphocytes (*p* < 0.01) in HPV+ve tumours (Fig. 1C; Supplementary Fig. 1G); also confirmed by MxIF (Fig. 1D). While HPV+ve tumours are largely characterised by an immune-hot tumour microenvironment, variation exists, with a proportion of HPV+ve tumours having lower TIL levels associated with poorer survival relative to TIL-high HPV+ve tumours [16]. Notably, there was a prominent fibroblast presence in both HPV-ve and HPV+ve tumours (Fig. 1C; Supplementary Fig. 1G).

A more detailed analysis of immune cell subsets (Supplementary Table 2) in the scRNA-Seq data revealed differences in T-cell and NK-cell phenotypes (56,664 cells) between HPV+ve and HPV-ve tumours (Supplementary Fig. 2A, B). *CD4* + *ICOS* + *PDCD1*+ (PD-1) T-cells, resembling T follicular helper (Tfh) cells were more common in HPV+ve tumours, as were CD4 + naïve-like T-cell clusters and *KIT +* NK-cells (Supplementary Fig. 2B). Analysis of B- and plasma cells (26,156 cells) showed that germinal centre (GC) B-cells (*RGS13*+, *NEIL1*+), cycling B-cells (*UBE2C*+, *TYMS*+) and naïve B-cells (*TCL1A*+, *IL4R*+) were all enriched in HPV+ve tumours compared to HPV-ve tumours (Supplementary Fig. 2C, D), while switched B-cell subsets were found in both. There were no differentially abundant myeloid populations (Supplementary Fig. 2E, F).

### scRNA-Seq of HNSCC reveals distinct subsets of inflammatory fibroblasts

**Fig. 2:**
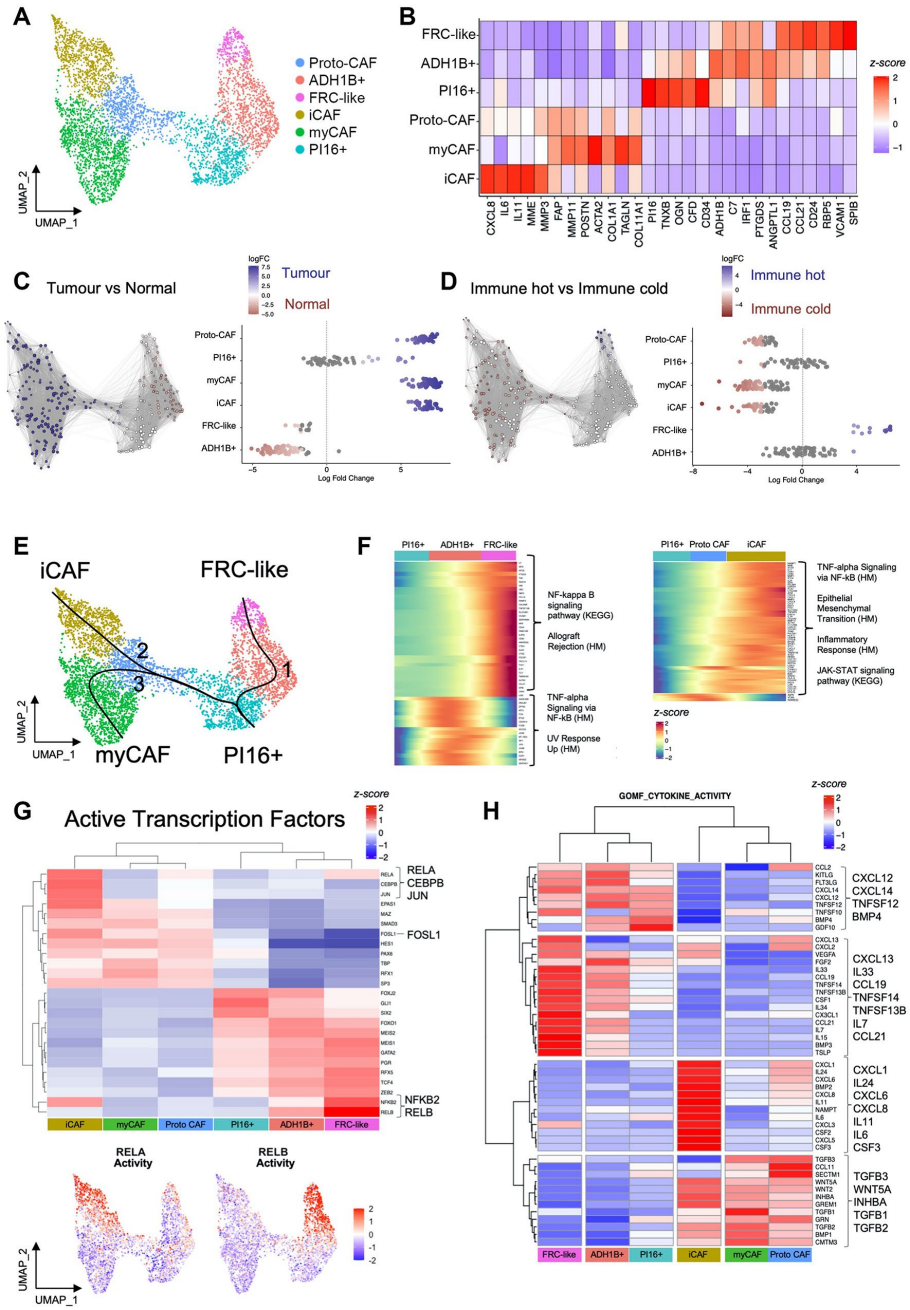
scRNA-Seq of HNSCC reveals distinct subsets of immunomodulatory fibroblasts. (**A**) UMAP of fibroblasts from integrated HNSCC dataset showing six clusters (4,894 cells; *n* = 24 HNSCC; *n* = 7 normal). (**B**) Heatmap showing the average expression of selected differentially expressed genes for each fibroblast cluster. (**C**) Differential abundance testing between HNSCC and normal samples. Highlighting differentially abundant neighbourhoods. (**D**) Differential abundance testing between immune-hot (*n* = 8) and immune-cold (*n* = 8) HNSCC. Samples classified based on lymphocyte content (see methods). (**E**) Trajectory analysis showing fibroblast lineages arising from universal (*PI16+*) fibroblasts. Lineage reconstruction and pseudotime inference using Slingshot package. (**F**) Examination of potential signalling pathways regulating iCAF and FRC-like inflammatory subsets by assessing pathway enrichment in genes that change as a function of pseudotime in the KEGG and Hallmarks gene sets (determined using Monocle 3 trajectory; q_value < 0.05 & morans_I > 0.25). Over-representation analysis showing selected enriched pathways of pseudotime-dependent genes. (**G**) Heatmap of activity of the top 25 transcription factors using DoRothEA regulons (wmean). Clustered scaled activity scores are shown. Below the heatmap shows scaled activity of RELA and RELB. (**H**) Average expression of genes with cytokine activity across fibroblast clusters. Differentially expressed genes filtered for GOMF_CYTOKINE ACTIVITY MSigDB gene set

To investigate fibroblast phenotypes present in HNSCC, we first broadly identified all fibroblasts based on lumican expression (*LUM+;* 4,894 cells); fibroblasts clustered closely with *RGS5 +* mural cells (2,174 cells), which included pericytes and smooth muscle cells (SMCs; Supplementary Fig. 3A, B). We identified six clusters of fibroblasts; three confined to tumours (CAF) and three present in both tumours and normal tissue (Fig. 2A; Supplementary Fig. 3C, D, E; Supplementary Table 3). Overall, individual patient tumours showed significant fibroblast heterogeneity, generally containing a mixture of the six phenotypes (Supplementary Fig. 3E, F).

The largest CAF cluster expressed canonical myofibroblastic CAF (myCAF) markers (*POSTN*, *MMP11*, *ACTA2*) and, as expected, showed highest enrichment for TGFβ signalling (Fig. 2B; Supplementary Fig. 3G). This cluster was characterised by high expression of ECM genes (including *COL1A1*, *FN1*, *COL1A2*, *COL6A3* and *COL11A1*); with numerous differentially expressed genes (DEGs) associated with core matrisome components [collagens (*n* = 14), glycoproteins (*n* = 22) and proteoglycans (*n* = 5)] (Supplementary Fig. 3H; Supplementary Table 3 [27]).

An inflammatory CAF (iCAF) population was characterised by high expression of inflammatory cytokines (e.g., *IL11*, *IL6*, *CXCL8*, *CXCL1*, *CXCL5*; Fig. 2B). Notably, iCAF also expressed upregulated ECM genes (albeit at a comparatively lower level than myCAF), with higher levels of genes associated with ECM remodelling (*MMP3*, *MMP1*, *PLAU*), glycolysis/hypoxia (*HIF1A*, *ENO1*, *GK*, *CA12*, *SLC16A3*/MCT4) and neutrophil-recruiting chemokines (*CXCL1*, *CXCL5*, *CXCL6*, *CXCL8)* (Supplementary Table 3). This cluster was highly enriched for hypoxia, MAPK, NF-κB, and TNFα signalling pathways (Supplementary Fig. 3G).

A further CAF cluster expressed lower levels of myCAF/iCAF marker genes; this ‘proto-CAF’ cluster displayed few unique DEGs (*n* = 19) compared with other fibroblast phenotypes (which ranged from *n* = 83–232 unique DEGs) and on the UMAP adjoined normal fibroblast and CAF clusters, likely representing a transition state. myCAF and iCAF were present in both HPV-ve and HPV+ve HNSCC (Supplementary Fig. 3E, F), but HPV-ve HNSCC samples contained greater proportions of CAF/fibroblasts relative to total cell number per sample (Supplementary Fig. 3E).

Within normal mucosa we identified three fibroblast subtypes (also present in tumours). Universal (adventitial) fibroblasts expressing *PI16* were present in normal mucosa, HPV+ve and HPV-ve tumour samples (Supplementary Fig. 3E, F). These expressed *CD34* and distinctive ECM-associated genes, including *COL14A1*, *OGN* and *TNXB*, likely reflecting their vascular-niche function (Fig. 2B, Supplementary Fig. 3H). *ADH1B +* fibroblasts were the most common subtype in normal tissue (54% of fibroblasts) but were infrequent in tumours (5% of fibroblasts; Fig. 2C; Supplementary Fig. 3E). The core matrisome profile of *ADH1B +* fibroblasts was similar to universal (*PI16+)* fibroblasts (Supplementary Fig. 3H). The third fibroblast subgroup expressed *CCL19*, *CCL21*, *VCAM1*, *RBP5* and *SPIB* (Fig. 2B) with a phenotype akin to fibroblastic reticular cells (FRC), specialised fibroblast subsets of lymphoid tissues that organise and traffic lymphoid cells.

Notably, when present in tumours, normal fibroblast subtypes up-regulated activation- (*FAP*, *FN1*, *PDPN*, *COL1A1*), inflammation- (*CXCL1*, *ISG15*) and insulin-like growth factor (IGF)-related (*IGF1*, *IGFBP2*, *IGFBP4*) genes (Supplementary Fig. 3I) suggesting early activation (with these genes expressed at higher levels in CAF clusters).

### Fibroblast phenotypes differ between immune-hot and immune-cold tumours

To investigate whether fibroblast subsets were differentially abundant in immune-hot and immune-cold tumours, we classified scRNA-Seq samples (*n =* 24) into ‘High’, ‘Moderate’ and ‘Low’ tertiles using lymphocyte abundance. Samples classified as lymphocyte-High were considered immune-hot tumours, of which all were HPV+ve, whereas lymphocyte-Low samples were considered immune-cold. FRC-like fibroblasts were the only fibroblast subset significantly enriched in immune-hot tumours (Fig. 2D), with 12/12 FRC-like neighbourhoods significantly differentially abundant (logfc > 3.75; spatialFDR < 0.05). Levels of FRC-like fibroblasts varied between individual HPV+ve tumours but were uniformly rare in all HPV-ve cases (Supplementary Fig. 3E, F, J). Immune-cold tumours were found to contain greater abundance of myCAF, iCAF and proto-CAF, with 60–65% of neighbourhoods within these clusters labelled as differentially abundant (logfc<-2.75; spatialFDR < 0.05).

### Regulation of the iCAF and FRC-like inflammatory phenotypes

We sought to identify how the different inflammatory fibroblast phenotypes were regulated by inferring lineages arising from universal (*PI16+*) fibroblasts [1]. To achieve this we performed trajectory analysis, which identified three lineages leading to the formation of FRC-like fibroblasts (through ‘*ADH1B+*’), myCAF and iCAF (both through ‘proto-CAF’; Fig. 2E; Supplementary Fig. 3K).

We next examined signalling pathways regulating FRC-like and iCAF inflammatory subsets by assessing pathway enrichment in genes changing as a function of pseudotime in KEGG and Hallmarks gene sets (q_value < 0.05 & morans_I > 0.25). Pseudotime analysis of the FRC-like lineage (1) showed increased expression of genes associated with NF-κB signalling pathway (KEGG; p.adjust < 0.01) and Allograft rejection (Hallmarks; p.adjust < 0.0001) (Fig. 2F; Supplementary Table 4). While many genes (e.g., *CCL19*, *C7*, *IRF8*) showed a pseudotime-dependent increase in expression through the *ADH1B +* cluster to FRC-like, other genes enriched for TNF-alpha Signalling via NF-κB (Hallmarks; p.adjust < 0.0001) increased in the *ADH1B +* cluster but decreased in the FRC-like cluster (e.g., *SOCS3*, *JUN*, *IRF1*, *FOS*, *JUNB*). Gene set enrichment analysis (GSEA) of the MSigDB Hallmarks gene sets revealed significant enrichment for allograft rejection in FRC-like fibroblasts (Supplementary Fig. 3L).

In the iCAF lineage (2), pseudotime analysis revealed increased expression of genes associated with TNF-alpha signalling via NF-κB (Hallmarks; p.adjust < 0.0001), Epithelial Mesenchymal transition (Hallmarks; p.adjust < 0.0001), inflammatory response (Hallmarks; p.adjust < 0.0001) and JAK-STAT signalling pathway (KEGG; p.adjust < 0.01) (Fig. 2F; Supplementary Table 4). GSEA showed enrichment for Glycolysis, Hypoxia, inflammatory response and TNF-alpha signalling via NF-κB (Supplementary Fig. 3L).

Intriguingly, while the chief inflammatory pathway, NF-κB, was associated with both iCAF and FRC-like inflammatory phenotypes, the corresponding genes differed. FRC-like NF-κB genes (e.g., *CCL21*, *CCL19*, *TNFSF13B*) are specifically associated with the alternative NF-κB pathway, commonly triggered through lymphotoxin, LIGHT, CD40-L and BAFF, and are related to lymphoid organ development and adaptive immunity [28]. Conversely, iCAF NF-κB genes (e.g., *CXCL1*, *CXCL8*) are generally associated with the classical NK-κB pathway, typically activated via IL-1, TNF-α or LPS and associated with inflammation and innate immunity [28]. Accordingly, we examined transcription factor (TF) activity in FRC-like and iCAF subsets by assessing the transcriptomic ‘footprint’ of active transcription factors using the DoRothEA database [23]. FRC-like fibroblasts showed strong activity for RELB and NFKB2 (p100/p52), again providing evidence for alternative NF-κB pathway activation through NF-κB RelB-p52 complexes, whereas top iCAF active transcription factors included RELA (p65), CEBPB and JUN/FOSL1 (AP1; Fig. 2G).

### Analysis of fibroblast phenotypes: spatial distribution, immune cell interactions and regulation of phenotypes

FRC-like fibroblasts and iCAF possessed distinct inflammatory cytokine profiles (Fig. 2H); FRC-like cytokines were associated with lymphocyte recruitment, proliferation, and survival [e.g., *CCL19/21*, *IL7/15*, *TNFSF14* (LIGHT), *TNFSF13B* (BAFF), *CXCL13*], with iCAF-specific cytokines related to myeloid/ granulocyte recruitment and differentiation (*CXCL1/5/6/8*, *CSF2/3*). This, in addition to their different regulatory pathways, suggested that FRC-like fibroblasts and iCAF were likely associated with distinct immunological niches. To investigate this, we first performed correlative sample-level analysis on scRNA-Seq data using the previously identified immune cell subsets in the integrated HNSCC scRNA-Seq dataset (Supplementary Fig. 2). We followed this with spatial transcriptomics (Visium 10x) analysis; using the annotated scRNA-Seq data as a reference to derive cell-type specific gene signatures that were used to deconvolute cell types present within each 55 μm spot [29, 30]. Applying this integrative approach for myCAF and universal (*PI16+*) fibroblasts, identified previously described spatial and cellular relationships (Supplementary Fig. 4).

### FRC-like fibroblasts are found within TLS and colocalise with B-cells and CD4 + T-cells

**Fig. 3:**
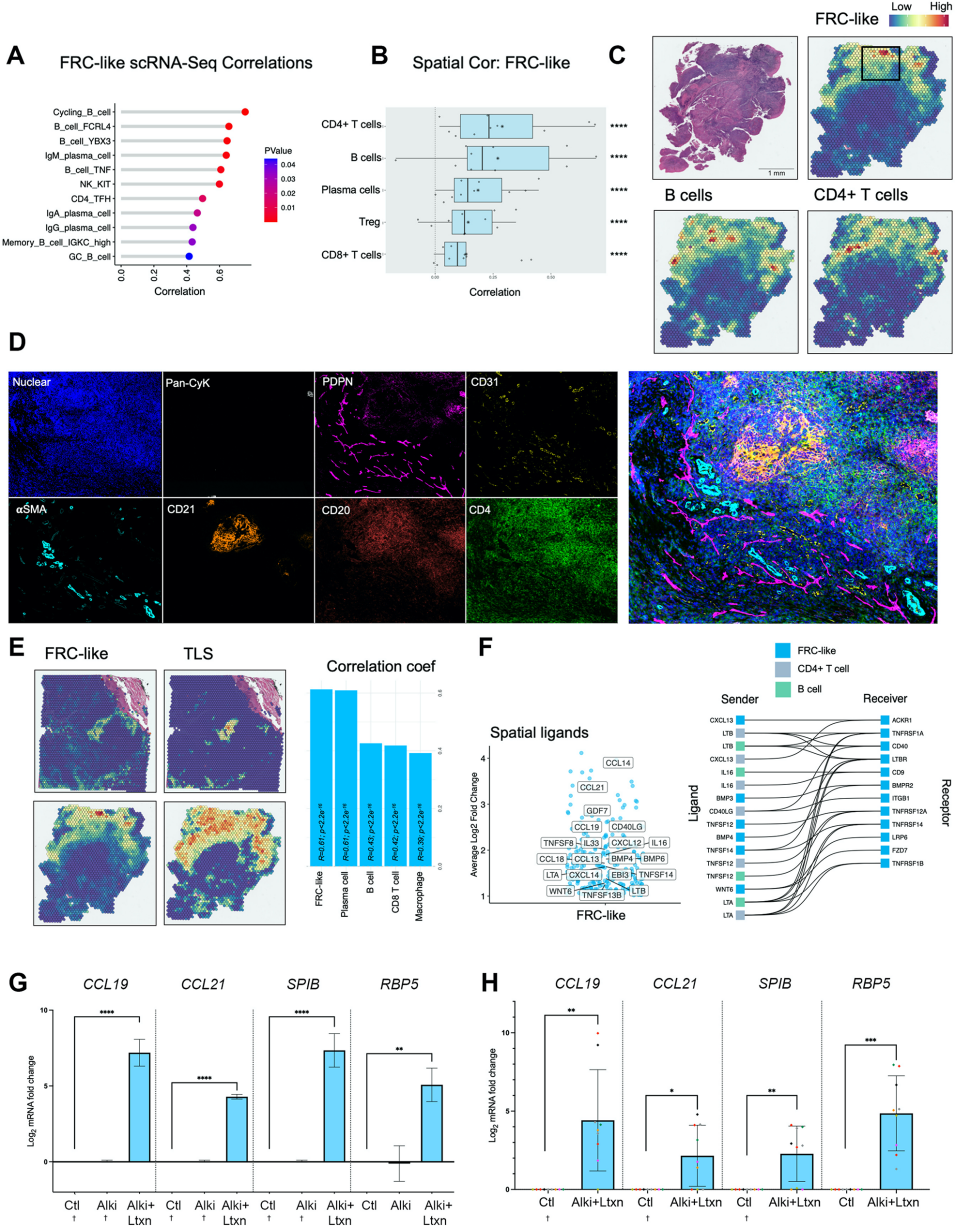
FRC-like fibroblasts colocalise with B-cells and CD4 + T-cells, found within TLS and are regulated via LTβR signalling. (**A**) FRC-like fibroblast and immune cell sample-level scRNA-Seq correlations (spearman; *p* < 0.05). For HNSCC samples only, fibroblast proportions (relative to total fibroblasts) per sample were correlated against immune cell cluster proportions (relative to total immune cells). Only significant positive associations are shown. (**B**) Spatial transcriptomics cell type correlations (spearman) using RCTD imputed abundance (normalised weights ≥ 0.05). Visium (10x) spots were deconvoluted using RCTD. Spearman correlation of normalised weights was carried out on each patient separately. Correlation coefficients are plotted for each of 10 patients, median displayed as vertical line in boxplot and mean as star symbol. Weighted Fisher’s method was used to combine p values. (**C**) Spatial feature plot of deconvoluted values of FRC-like fibroblasts, B-cells and CD4 + T-cells in a HPV + ve HNSCC sample. (**D**) MxIF (Phenocycler-Fusion) showing staining (DAPI, Pan-cytokeratin, PDPN, CD31, αSMA, CD21, CD20, CD4) in FRC-like containing region of interest identified by RCTD deconvolution. MxIF markers are shown separately and accompanied by composite image of all markers. PDPN + CD31- cells marking fibroblasts. (**E**) FRC-like abundance (RCTD) and TLS signature [33] enrichment (AddModuleScore) spatial feature plot with spearman correlation of RCTD normalised weights. Correlations for each Visium (10x) spot across all 10 patients. Top 5 correlations shown, including FRC-like fibroblasts with highest correlation coefficient. (**F**) Volcano and ligand-receptor interaction plots showing spatially differentially expressed ligands (Log2FC ≥ 1; padj < 0.0001). Differentially expressed ligands in FRC-like containing spots (normalised weight ≥ 0.05). Ligands displayed are those found within the GOMF_CYTOKINE_ACTIVITY MSigDB gene set; expressed in FRC-like fibroblasts, B-cells or CD4 + T-cells; and have expression of corresponding receptor in FRC-like fibroblasts. (**G**) qPCR analysis of FRC-like fibroblast markers (*CCL19*,* CCL21*,* SPIB and RBP5*) in primary NOF treated with a TGFBR1 inhibitor (ALKi; 1µM) and 50ng/ml LTa1β2 for 7 days. Results show mean ± SD of 3 independent experiments in *n* = 1 primary NOF cell line. One-way ANOVA with Bonferroni correction. (**H**) qPCR analysis of FRC-like fibroblast markers (*CCL19*,* CCL21*,* SPIB and RBP5*) in *n* = 7 primary NOF lines treated with 100ng/ml LTa1β2 + ALKi (1µM) for 48 h. Results show mean ± SD of 9 independent experiments, colours of points correspond to primary NOF line. Paired Student t test (two-tailed). † = Ct undetermined, assumed Ct = 40. *p < 0.05; **p < 0.01; ***p < 0.001. ****p < 0.0001

In sample-level scRNA-Seq correlations of tumours, FRC-like fibroblasts positively correlated with various B-cell subsets [including cycling B-cells, *FCRL4* + B-cells and germinal centre (GC) B-cells], plasma cells, *KIT +* NK-cells (Fig. 3A), with high correlation with IgM expressing B/plasma cells. FRC-like fibroblasts also correlated with CD4 + T follicular helper (Tfh) cells. Spatial transcriptomic analysis confirmed that each tumour contained multiple fibroblast subsets that were spatially discrete (82.4% of fibroblast-containing spots contained one subset only; Supplementary Fig. 5). FRC-like fibroblasts colocalised with B-cells and CD4 + T-cells (non-Treg; *p* < 0.0001), found either in focal areas containing high densities of B/CD4 + cells (non-Treg) (HPV+ve/-ve HNSCC) or occasionally more widespread in two HPV+ve samples (59%/23% total spots) but still colocalising with large numbers of B/CD4 + T-cells (Fig. 3BC; Supplementary Fig. 6A, B, C). There was also a spatial correlation between FRC-like fibroblasts and plasma cells, Tregs and CD8 + T-cells (Fig. 3B; Supplementary Fig. 6A, B). PDPN is commonly utilised as a pan-fibroblast marker and has been specifically employed to identify FRC in lymph nodes [31]. MxIF on spatial transcriptomic-determined FRC-like regions of interest (ROI) confirmed the presence of PDPN+/CD31- fibroblasts colocalising with CD20 + and CD4 + cells (Fig. 3D; Supplementary Fig. 6D) within and surrounding CD21 + follicular dendritic cells, along with high densities of B-cells, suggesting formation of tertiary lymphoid structures (TLS). We therefore compared TLS and FRC-like enrichment in spatial transcriptomic data using different TLS gene signatures [32]. Spot deconvolution (RCTD) showed that FRC-like fibroblasts significantly correlated with module enrichment scores for various TLS signatures that have been used in several solid cancer studies (Spearman’s *r* ≥ 0.5, *p* < 0.0001; Fig. 3E, Supplementary Fig. 6E; [33–36]).

### FRC-like fibroblasts are regulated via LTβR signalling

We next investigated potential interactions in the FRC-like niche by examining ligands and receptors that were differentially expressed in Visium spots that contained FRC-like fibroblasts (spots containing at least 5% FRC-like cells imputed by RCTD deconvolution; Supplementary Fig. 7A; Supplementary Table 5). LTβR-binding ligands [*TNFSF14* (LIGHT), *LTB*, *LTA*, *CD40LG*] were amongst cytokine activity-possessing ligands that were spatially associated, expressed by highly correlating cell types (Fig. 3F; Supplementary Fig. 7B) and known to stimulate alternative NF-κB pathway activation (consistent with previous pathway/TF analysis). LIGHT and CD40LG were top ligands inferred via NicheNet [37] (Supplementary Fig. 7C). Lymphotoxin was highly expressed in B-cells, T-cells and DCs (Supplementary Fig. 7D), whereas LIGHT was expressed by FRC-like fibroblasts. In common with FRC-like fibroblasts, most other fibroblast subsets also expressed receptors for these (and other) inflammatory ligands (Supplementary Fig. 7E), highlighting the potential for immunological plasticity in these cells depending on ligand availability.

We then assessed the ability of the LTβR-binding ligand lymphotoxin α1β2 (LT) to regulate the FRC-like phenotype in cultured primary oral fibroblasts (NOFs). Treatment with LT in combination with an ALK5 (TGFBR1) inhibitor induced FRC-like-specific genes *CCL19* (*p* < 0.0001), *CCL21* (*p* < 0.0001), *SPIB* (*p* < 0.0001), *RBP5* (*p* < 0.01; Fig. 3G). This was confirmed in further primary NOF cultures (*n* = 7; *CCL19*, *p* < 0.01; *CCL21*, *p* < 0.05; *SPIB*, *p* < 0.01; *RBP5*, *p* < 0.001; Fig. 3H).

### iCAF colocalise with inflammatory monocytes and neutrophils

**Fig. 4:**
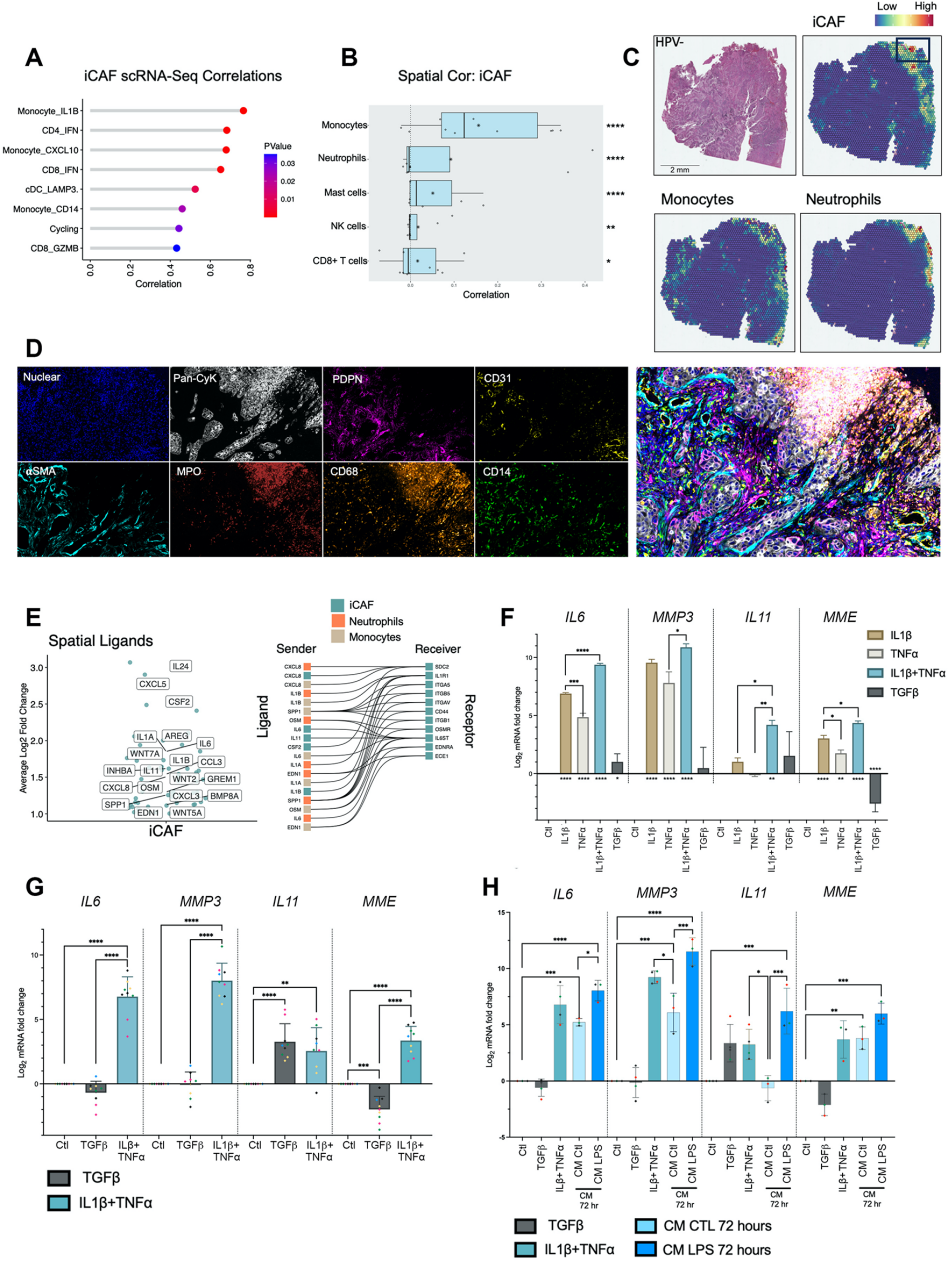
iCAF colocalise with inflammatory monocytes and neutrophils and are regulated via IL-1β and TNF-α signalling. (**A**) iCAF and immune cell sample-level scRNA-Seq correlations (spearman; *p* < 0.05). For HNSCC samples only, fibroblast proportions (relative to total fibroblasts) per sample were correlated against immune cell cluster proportions (relative to total immune cells). Only significant positive associations are shown. (**B**) Spatial transcriptomics cell type correlations (spearman) using RCTD imputed abundance (normalised weights ≥ 0.05). Visium (10x) spots were deconvoluted using RCTD. Spearman correlation of normalised weights was carried out on each patient separately. Correlation coefficients are plotted for each of 10 patients, median displayed as vertical line in boxplot and mean as star symbol. Weighted Fisher’s method was used to combine p values. (**C**) Spatial feature plot of deconvoluted values of iCAF, monocytes and neutrophils in a HPV-ve HNSCC sample. (**D**) MxIF (Phenocycler-Fusion) showing staining (DAPI, Pan-cytokeratin, PDPN, CD31, αSMA, MPO, CD68, CD14) in iCAF containing region of interest identified by RCTD deconvolution. MxIF markers are shown separately and accompanied by composite image of all markers. (**E**) Volcano and ligand-receptor interaction plots showing spatially differentially expressed ligands (Log2FC ≥ 1; padj < 0.0001). Differentially expressed ligands identified using FindMarkers on iCAF containing spots (normalised weight ≥ 0.05) filtered for ligands. Ligands displayed are those found within the GOMF_CYTOKINE_ACTIVITY MSigDB gene set; expressed in iCAF, monocytes or neutrophils; and have expression of corresponding receptor in iCAF. (**F**) qPCR analysis of iCAF markers (*IL6*, *MMP3*, *IL11* and *MME*) in primary NOF treated with IL1β (1ng/mL), TNF𝛼 (1ng/mL), IL1β (1ng/mL) + TNF𝛼 (1ng/mL) and TGFβ (4ng/mL) for 48 h. Results show mean ± SD of 3 biological replicates in *n* = 1 primary NOF cell line. One-way ANOVA with Bonferroni correction. P values marked by asterisk under bars reflect comparisons with CTL. (**G**) qPCR analysis of iCAF markers (*IL6*, *MMP3*, *IL11* and *MME*) in *n* = 5 primary NOF lines treated with TGFβ (4ng/mL) or IL1β (1ng/mL) + TNF𝛼 (1ng/mL) for 72 h. Results show mean ± SD of *n* = 9 independent experiments, colours of points correspond to primary NOF line. One-way ANOVA with Bonferroni correction. (**H**) qPCR analysis of iCAF markers (*IL6*, *MMP3*, *IL11* and *MME*) in *n* = 3/4 primary NOF lines treated with TGFβ (4ng/mL), IL1β (1ng/mL) + TNF𝛼 (1ng/mL), monocyte conditioned media (CM) or LPS-activated monocyte conditioned media for 72 h. Results show mean ± SD of *n* ≥ 3 independent experiments, colours of points correspond to primary NOF line. One-way ANOVA with Bonferroni correction. *p < 0.05; **p < 0.01; ***p < 0.001. ****p < 0.0001

iCAF correlated strongly with a subset of *CD14* + *IL1B*^*high*^ inflammatory monocytes (Spearman’s *r* = 0.72, *p* < 0.0001; Fig. 4A; Supplementary Fig. 8A). Spatial transcriptomic analysis showed that iCAF were spatially distinct from myCAF (Supplementary Fig. 5), located primarily at the tumour periphery, particularly towards the tumour surface. iCAF colocalised with monocytes and neutrophils (*p* < 0.0001), also frequently found at the periphery of tumours (Fig. 4B, C; Supplementary Fig. 6A, B; Supplementary Fig. 8C). MxIF on iCAF ROI (identified through spatial transcriptomics deconvolution) showed that these areas contained PDPN+/CD31- fibroblasts and were associated with disruptions in surface epithelium (pan-cytokeratin) and CD68+, CD14 + and MPO + myeloid cells (Fig. 4D; Supplementary Fig. 8C).

### iCAF are regulated via IL-1β and TNF-α

Next, we examined the expression of spatially located ligands in cell types highly correlating with iCAF (Supplementary Table 5). Top ligands associated with cytokine activity in the iCAF-niche included *CXCL8*, *IL1A*, *IL6*, *OSM*, *IL11* and particularly *IL1B* which was highly expressed by inflammatory monocytes (Fig. 4E; Supplementary Fig. 8D). We also inferred ligand regulatory activity using NicheNet, which highlighted IL1B and IL1A as top spatially defined ligands with iCAF (gene set) regulatory potential (Supplementary Fig. 8E). Indeed, myeloid cells (monocytes, neutrophils, macrophage) expressed highest levels of these ligands (*IL1B*, *IL1A*, *OSM*), supportive of iCAF associating with a myeloid niche (Supplementary Fig. 7D).

We tested the potential of these ligands for regulating the iCAF phenotype in NOFs. In addition to IL-1β, we included TNF-α due to pathway and TF enrichment for classical NF-κB signalling and high expression in monocytes [although *TNF* was not spatially differentially expressed in the iCAF niche, likely resulting from expression in multiple immune cell types (Supplementary Fig. 7D)]. NOFs were treated with IL-1β and TNF-α, either alone or in combination. We also included TGF-β1, a central regulator of the myCAF phenotype for reference. Both IL-1β and TNF-α induced expression of *IL6* (*p* < 0.0001), *MMP3* (*p* < 0.0001) and *MME* (*p* < 0.01; Fig. 4F), but with limited upregulation of *IL11*. However, combining IL-1β with TNF-α increased expression of all inflammatory marker genes compared with individual treatments (*IL6*, *p* < 0.0001; *MME*, *p* < 0.05), including *IL11*, which increased 16-fold (log_2_FC = 4) compared to IL-1β alone (*p* < 0.05). This was validated in several primary fibroblast cultures (*n* = 9), where TNF-α and IL-1β robustly induced iCAF gene expression (Fig. 4G). *IL11* was also induced by TGF-β1 (*p* < 0.0001); conversely, *ACTA2* (αSMA; a myCAF marker) was induced by TNF-α/IL-1β (*p* < 0.001); while other iCAF (*IL6*, *MMP3*, *MME*) and myCAF (*POSTN*, *TAGLN*, *COL1A1*) genes were more specifically regulated by TNF-α/IL-1β and TGF-β1 respectively (Supplementary Fig. 8F).

Given the iCAF/monocyte spatial relationship, we investigated whether monocytes regulated the iCAF phenotype. NOF treated with conditioned medium from monocytes activated with LPS induced upregulated expression of iCAF genes (*IL6*, *p* < 0.0001; *MMP3*, *p* < 0.0001, *IL11*, *p* < 0.001; *MME*, *p* < 0.001; Fig. 4H). Similar to TNF-α/IL-1β treatment, *ACTA2* was also increased (*p* < 0.05; Supplementary Fig. 8G).

In summary, the two immune-related HNSCC fibroblast subtypes occupy distinct immunological niches; FRC-like are found in TLS with CD4 + T-cells and B-cells and regulated through non-canonical NF-κB signalling via LTBR; iCAF are found with inflammatory monocytes and neutrophils and regulated through canonical NF-κB signalling through TNF-α and IL-1β.

### Pan-cancer fibroblast analysis identifies conserved and semi-conserved inflammatory fibroblast phenotypes

**Fig. 5:**
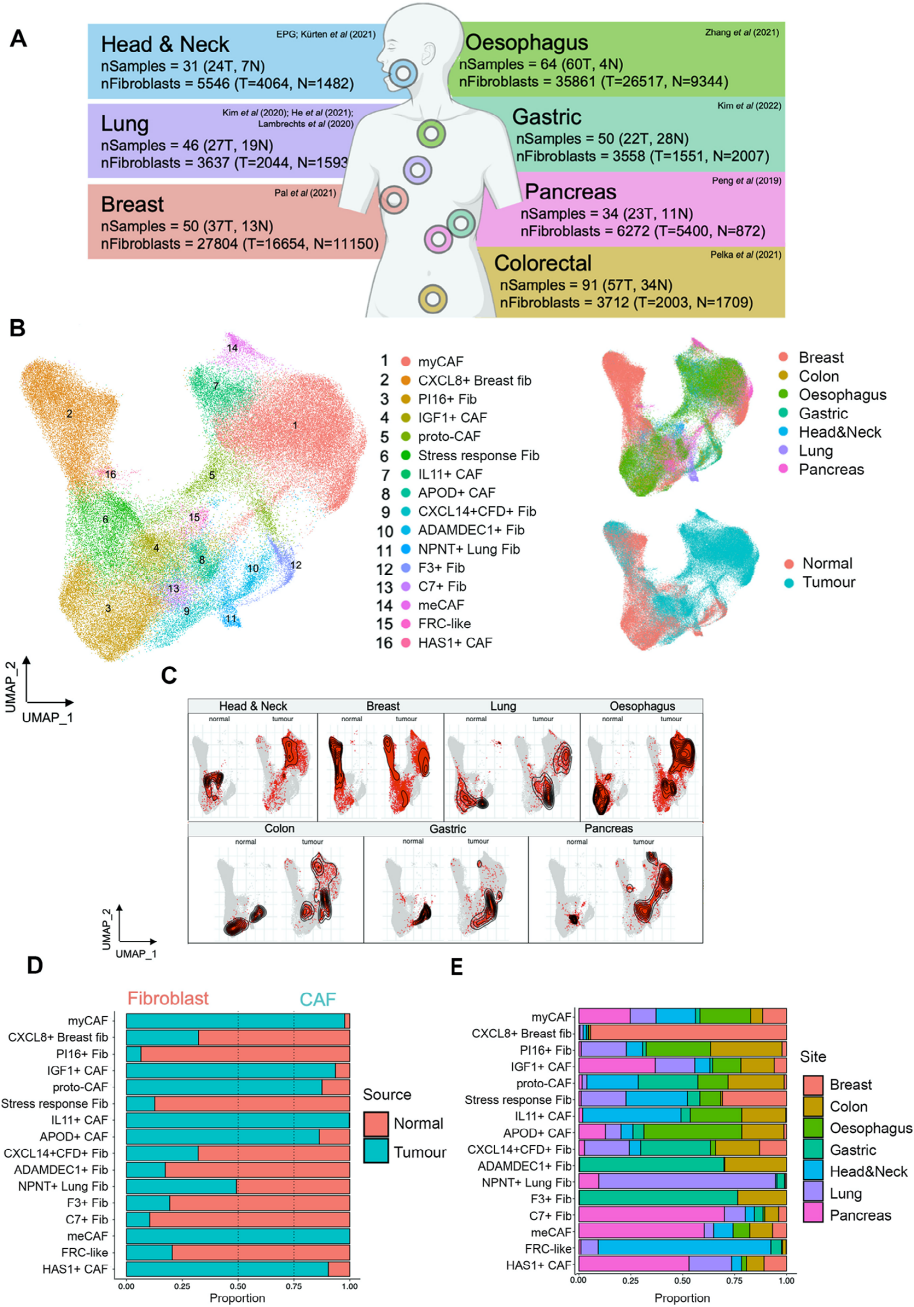
Pan-Cancer fibroblast analysis identifies conserved and semiconserved inflammatory fibroblast phenotypes. (**A**) Schematic of Pan-Cancer Fibroblast Atlas (PCFA) including anatomical sites, sample/fibroblast numbers and original publications. This integrated PCFA contained 86,414 fibroblasts from 376 samples. (**B**) UMAP plot of PCFA displaying 16 clusters, and to the right, UMAPs coloured by anatomical site and source of sample (tumour or normal). Samples were integrated using harmony via Seurat v5 sketch-based integration. (**C**) PCFA UMAP split by anatomical site and tumour/normal samples with density of fibroblasts highlighted on UMAP. (**D**) Relative proportion of each cluster in down-sampled (to same number of cells from each anatomical site and same number of cells from tumour/normal samples) normal and tumour samples. (**E**) Relative proportion of each cluster in down-sampled (to same number) anatomical sites (including normal and tumour samples)

To compare the HNSCC fibroblast phenotypes with other cancers, we generated a scRNA-Seq (10x Chromium) pan-cancer fibroblast atlas (PCFA) from seven cancer types: HNSCC, pancreatic, breast, lung, colon, oesophageal and gastric cancers (Fig. 5A). Only datasets containing both tumour and normal samples were included to differentiate between normal (steady-state) and cancer-associated phenotypes (Fig. 5B, C, D).

The PCFA (86,414 fibroblasts; 376 samples), revealed 16 populations, including broadly conserved, as well as tissue-specific subsets. Where possible, fibroblast subgroups were labelled using designations from previous studies (Supplementary Table 6). Conserved phenotypes in normal tissue included universal (*PI16+*) Fib, stress-response Fib (*DNAJB1+*, *HSPH1+*, *HSPA1A+;* which incorporated the head & neck *ADH1B +* fibroblasts) and *CXCL14 + CFD +* Fib (Fig. 5E). Tissue-specific subsets included *CXCL8* + breast fibroblasts [most abundant subset (>85%) in normal breast tissue], *NPNT*+ (alveolar) lung fibroblasts and *F3*+/*ADAMDEC1* + colonic/gastric fibroblasts (all found in normal and tumour samples; Fig. 5D, E). The FRC-like cluster was mostly composed of cells from the head & neck with a contribution from lung (from normal tissues and cancers) and low numbers from other cancer types (Fig. 5D, E).

Only subsets found exclusively in cancers were termed CAF (Fig. 5D). Conserved common CAF clusters included myCAF (the most abundant CAF subset in all tumour types except gastric cancer; Supplementary Fig. 9A), *IGF1 +* CAF and proto-CAF. Other clusters had increased frequency in certain cancer types or were rare across cancers. For example, three clusters had a larger contribution from pancreatic tumour/normal samples (*HAS1 +* CAF, metabolic CAF [meCAF] and *C7 +* Fib (Fig. 5E; Supplementary Fig. 9A). meCAF expressed markers of glycolysis and hypoxia (*ENO1*, *ENO2*, *NDRG1*, *PGK1*, *LDHA*, *SLC2A1*) consistent with a previously described population [38]. Although this cluster was observed pan-cancer, outside of pancreatic samples it was generally found at very low number (Supplementary Fig. 9A). No *bona fide* apCAF subpopulation was detected, but *CD74* was differentially expressed in several clusters (Supplementary Table 6).

The HNSCC iCAF resided in the *IL11 +* CAF cluster (Supplementary Fig. 9B); this semi-conserved phenotype was one of the most abundant fibroblast subpopulations in HNSCC, colorectal carcinoma (CRC) and oesophageal squamous cell carcinoma (ESCC) (Supplementary Fig. 9A) but was not present in lung or breast cancers. Label transfer using the HNSCC myeloid cells as a reference to identify myeloid phenotypes in CRC/ESCC scRNA-Seq datasets showed that *IL11 +* CAF similarly correlated with *IL1B +* inflammatory monocytes suggesting the same immunological niche is present in different cancer types (Supplementary Fig. 9C).

### ‘iCAF’ gene signature highlights different normal fibroblast and CAF populations

**Fig. 6:**
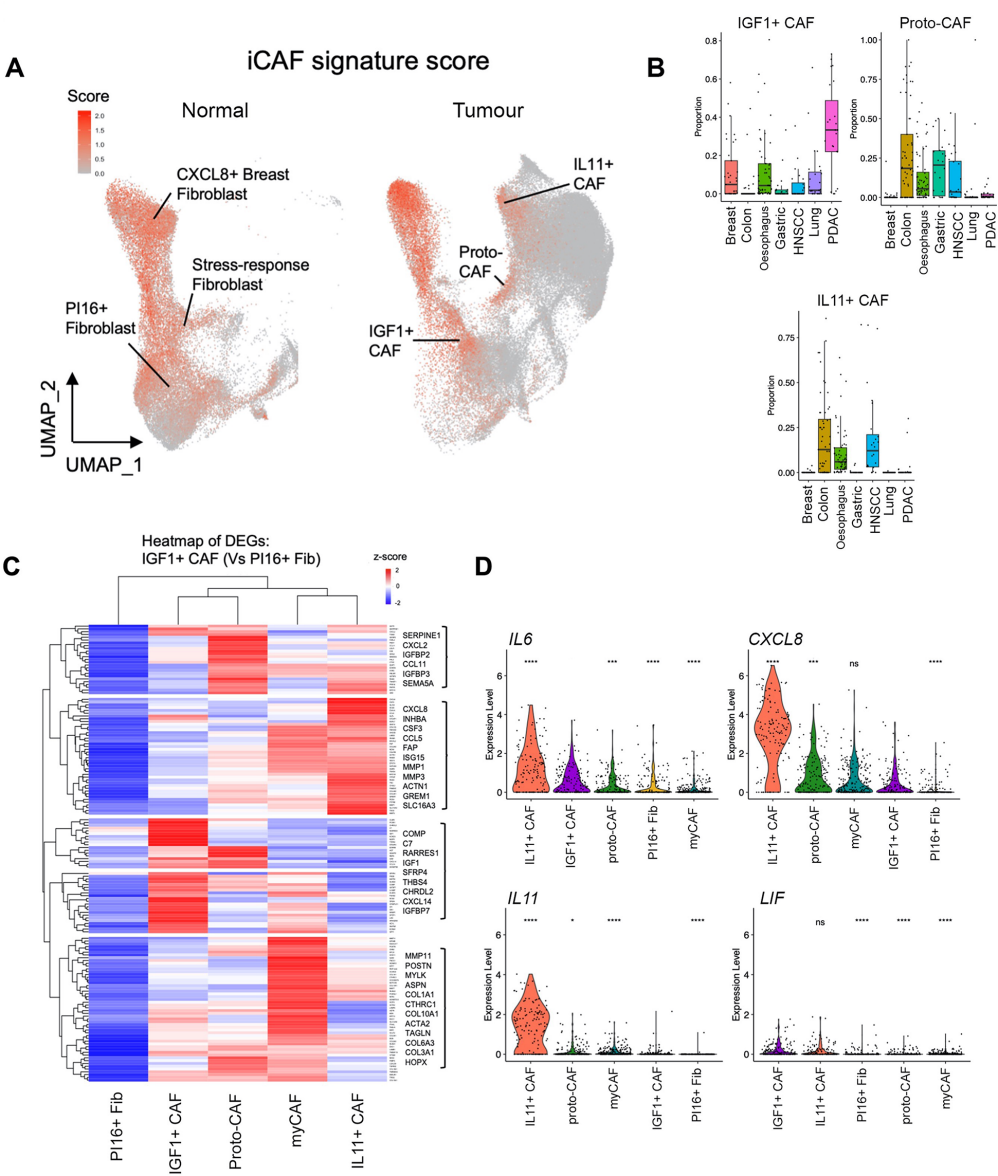
‘iCAF’ gene signature highlights different normal fibroblast and CAF populations. (**A**) Feature plot (UMAP) showing expression of iCAF signature enrichment in PCFA, split by tumour or normal samples. AddModuleScore using the 12-gene iCAF signature from Elyada et al., (2019) [5]. (**B**) Proportion of *IL11 +* CAF, proto-CAF and *IGF1 +* CAF in tumour samples across cancer types. (**C**) Heatmap showing average expression of DEGs upregulated in *IGF1 +* iCAF compared to universal (*PI16+*) fibroblasts. Clustering of rows form gene modules. (**D**) Selected iCAF gene (*IL6*, *CXCL8*, *IL11*, *LIF*) expression across clusters (sample-level). Wilcoxon rank-sum test (two-sided) compared to *IGF1* + CAF. ns *p* ≥ 0.05, *p < 0.05; **p < 0.01; ***p < 0.001. ****p < 0.0001

iCAF have been identified in several cancer types included in the PCFA (including PDAC and breast cancer), yet the iCAF population identified in HNSCC (*IL11* + CAF) was restricted to HNSCC and GI cancers. We therefore performed enrichment analysis using a previously described PDAC iCAF gene signature [5] to investigate whether other PCFA subgroups expressed previously described iCAF markers. This approach highlighted several putative iCAF-enriched populations from both normal and tumour samples; in normal tissues these included Stress-response Fib, *CXCL8 +* Breast Fib and universal (*PI16+*) fibroblasts; in tumours, *IL11 +* CAF, *IGF1 +* CAF and proto-CAF (Fig. 6A). We examined the iCAF signature-enriched clusters from tumours in more detail.

Notably, *IGF1 +* CAF expressed all PDAC iCAF markers suggesting this phenotype represented the iCAF described in PDAC (and breast cancer) studies (Supplementary Fig. 9D; [5, 6, 17, 18]). *IGF1 +* CAF were present in most cancer types (e.g., breast, oesophageal, lung), including high levels in PDAC (where they were comparable to myCAF in abundance; Fig. 6B; Supplementary Fig. 9A). Transcriptionally, *IGF1 +* CAF differed considerably from *IL11 +* CAF, clustering close to universal (*PI16+*) Fib and maintaining expression of universal (*PI16+*) Fib genes (*PI16*, *CFD*, *COL14A1*; Supplementary Table 7 A).

In our initial HNSCC analysis, ~ 50% of the (universal) *PI16 +* fibroblast cluster from tumour samples were labelled as *IGF1 +* CAF in the larger PCFA dataset. This showed that universal (*PI16+*) fibroblasts from tumours show evidence of activation and inflammatory changes (including expression of *FAP*, *COL1A1*, *IGF1*; Supplementary Fig. 3I; Supplementary Fig. 9B). Given the transcriptional similarity between universal (*PI16+*) fibroblasts and *IGF1 +* CAF, we examined DEGs between these phenotypes (Supplementary Table 7B). 159 DEGs (log2FC > 1; p.adj < 0.05) were identified as up-regulated in *IGF1* + CAF compared to *PI16 +* fibroblasts, including previously described markers of both iCAF and myCAF phenotypes (Supplementary Table 7B). We then analysed these DEGs across other common CAF populations (Fig. 6C). This showed 52/159 clustered DEGs were more strongly upregulated in myCAF (including *POSTN*, *MMP11*, *COL1A1*); and 42/159 clustered DEGs were more strongly up-regulated by the *IL11 +* CAF population, including key inflammatory cytokines (*CXCL8*, *CXCL2*, *CCL5*). Moreover, iCAF-associated cytokines *IL6*, *CXCL8 and IL11* (all *p* < 0.0001) were expressed markedly higher in *IL11 + CAF* compared to *IGF1 + CAF* (Fig. 6D).

These data suggest that *IGF1* + CAF are an early activated state and are likely the predominant iCAF described in the current literature. In comparison, *IL11* + CAF subtype are more inflammatory and found within cancers of the GI tract.

### FRC-like fibroblasts are present across cancers at low frequency and are associated with positive response to immunotherapy

**Fig. 7:**
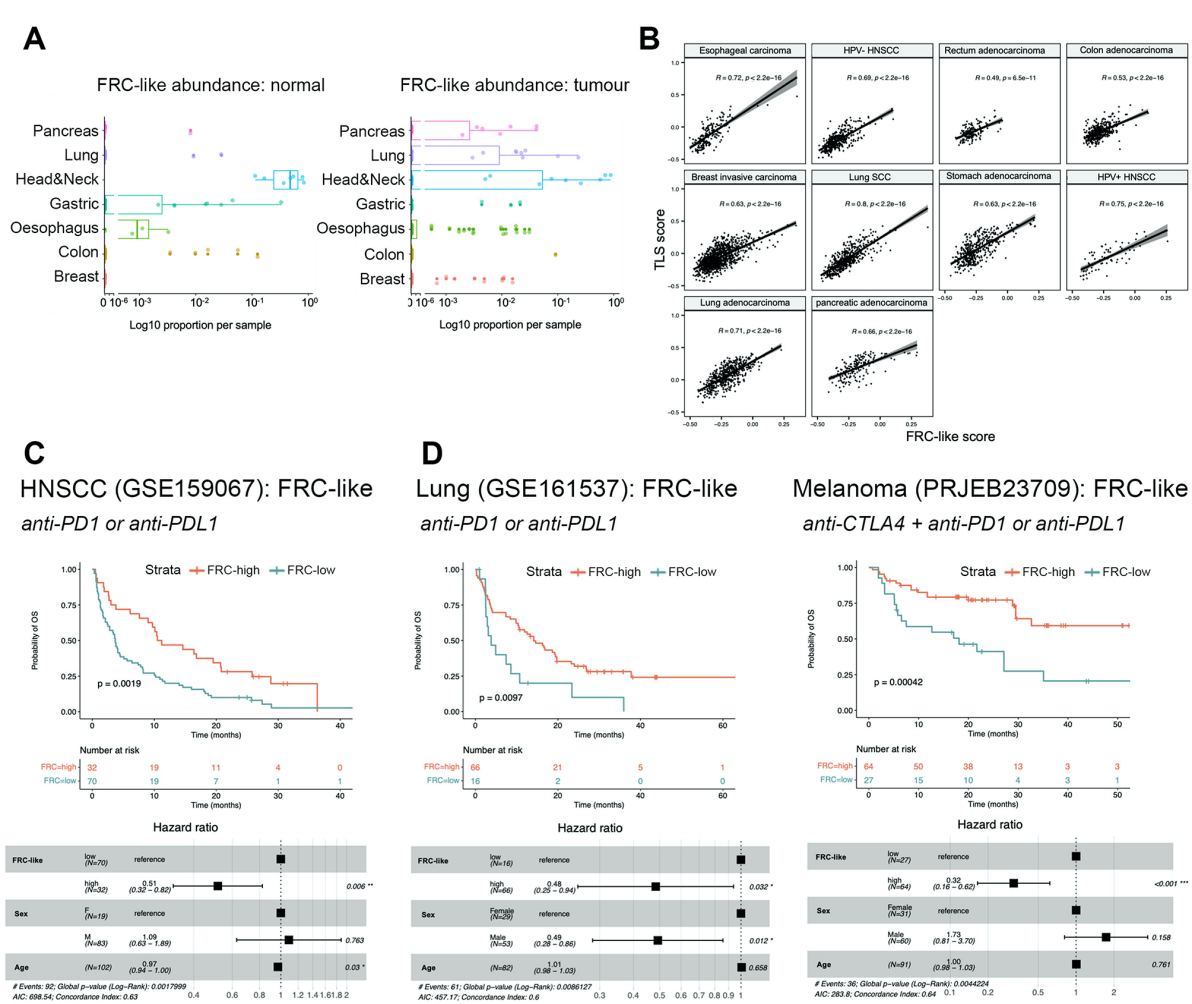
FRC-like fibroblasts are present across cancers at low frequency and are associated with positive response to immunotherapy. (**A**) Abundance of FRC-like fibroblasts across anatomical sites (normal only) and cancer types (tumour only). Log10 scale used due to extremely low abundance of FRC-like fibroblasts in non-head & neck tissue/tumours. (**B**) Correlation of FRC-like fibroblast signature and TLS signature [33] enrichment across selected cancer types in TCGA Bulk RNA-Seq data. ssGSEA run using batch effects normalized mRNA data from the Pan-Cancer Atlas Hub (UCSCXena). Spearman correlation coefficients and p-values displayed. (**C**) Kaplan-Meier (overall) survival plot showing anti-PD-1/PD-L1 treated HNSCC cohort (GSE159067; *n* = 102), stratified by FRC-like fibroblast (ssGSEA) scores. Below, forest plot for multivariate cox regression model using FRC-like level (high or low), patient sex and patient age. Hazard ratio estimates along with confidence intervals (95%) and p-values are plotted for each variable. (**D**) Kaplan-Meier (overall) survival plot showing anti-PD-1/PD-L1 treated NSCLC cohort (GSE161537; *n* = 82) and anti-CTLA-4 + anti-PD-1 or anti-PD-1 treated melanoma cohort (PRJEB23709; *n* = 91) stratified by FRC-like fibroblast (ssGSEA) scores. Below, forest plot for multivariate cox regression model using FRC-like level (high or low), patient sex and patient age. Hazard ratio estimates along with confidence intervals (95%) and p-values are plotted for each variable. Statistical significance shown on Kaplan-Meier plot assessed using a log-rank test

Across the seven cancers included in the PCFA, FRC-like cells were a relatively rare fibroblast population, with 77.8% of cells contributed from head and neck (62.1% normal; 15.6% tumour). Within tumour samples, FRC-like fibroblasts were found with highest average relative abundance in HNSCC (11.2%) followed by lung cancer (1.7%) but were present in all cancers at low frequencies (Fig. 7A). FRC-like cells were detected in several normal tissue sites, likely representing mucosa-associated lymphoid tissues (MALT; Fig. 7A). In breast, oesophageal, lung and pancreatic cancers, FRC-like fibroblasts were enriched in tumour samples, while were less common in HNSCC, gastric and colon cancer relative to normal samples.

The positive correlation between the FRC-like fibroblast and TLS gene signatures [33] across bulk RNA-Seq datasets (Fig. 7B), suggested that an FRC-like-containing immune hub exists in different cancers. Given that TLS have been linked with positive response to checkpoint immunotherapy in several cancer types [32, 33, 39] we investigated whether FRC-like fibroblasts were similarly associated. First, we utilised a dataset (GSE159067) consisting of pre-treatment samples from 102 patients with advanced HNSCC treated with anti-PD-1/PD-L1 immunotherapy. Samples were scored using ssGSEA for fibroblast subset-specific genes (Supplementary Table 8). We found patients with higher FRC-like scores had significantly improved survival (*p* < 0.01) (Fig. 7C). In contrast, iCAF were associated with significantly poorer survival (Supplementary Fig. 9E). We performed the same analysis on datasets from lung cancer and melanoma patients. Similarly, we observed higher FRC-like scores were associated with significantly improved survival in immune checkpoint inhibitor treated patients with lung cancer (*p* < 0.01; anti-PD1/PD-L1) and melanoma (*p* < 0.001; anti-CTLA4/PD-1); unlike HNSCC, iCAF were not prognostic (Fig. 7D; Supplementary Fig. 9F, G, H).

## Discussion

We characterised immune-hot and immune-cold HNSCC to investigate fibroblast phenotypes associated with the distinct immunological environments of HPV+ve and HPV-ve tumours, hypothesising that the high-TIL containing HPV+ve subset may contain a fibroblast phenotype that supports anti-tumour immunity. We included normal mucosa to separate cancer-associated phenotypes from those present in steady-state, as this was not included in recent HNSCC scRNA-Seq datasets [26, 40]. Single cell analysis identified six major fibroblast subgroups; universal *(PI16+*) fibroblasts, *ADH1B* + fibroblasts and *CCL19 + *FRC-like fibroblasts were present in normal tissue and tumours. myCAF, [*IL11 +*] iCAF and proto-CAF were limited to tumours. Of these, proto-CAF fibroblasts were likely a transition state as cells differentiated towards myCAF/iCAF phenotypes. HPV+ve and HPV-ve cancers contained mixtures of all fibroblast subgroups, although proportions and relative abundance varied in individual tumours. In support of our hypothesis, we found significantly higher numbers of FRC-like fibroblasts expressing *CCL19* and *CCL21* in immune-hot HPV+ve cancers. These tumours were situated in the oropharynx, an anatomical site that contains secondary lymphoid organs (SLO; tonsils) and considerable numbers of FRC-like cells were also present in matched normal oropharyngeal tissue. However, scRNASeq did not identify FRC-like fibroblasts in HPV-ve HNSCC tumours at the same site, or in a minority of HPV+ve tumours, suggesting either that FRC-like fibroblasts are retained within most HPV+ve tumours or arise *de novo*.

In lymph nodes, FRC play a central role in structural organisation; attracting and maintaining T-cells, supporting B-cell survival, promoting dendritic cell migration, and controlling permeability of high endothelial venules [31]. Similar cells arise in autoimmune disease, where they transdifferentiate from local fibroblasts and play a central role in supporting TLS formation and maintenance [41]. Consistent with this, within tumours we found FRC-like fibroblasts located with B-cells and (Tfh) CD4 + T-cells in TLS structures, correlating with several well-described TLS gene signatures. Development of mature FRCs from precursor cells in SLO is driven by LTβR signalling [31], and TLS-forming FRC-like ‘immunofibroblasts’ have been shown to be regulated by LTα1b2 and IL22 in Sjogren’s syndrome [41]. We found FRC-like fibroblasts to be similarly regulated through non-canonical NF-κB signalling, showing strong activity for transcription factors RELB and NKFB2 (p100/p52). LTβR signals via alternative NF-κB, binding to ligands such LTα1β2 and LIGHT [42]. *LTA*, *LTB*, LIGHT/*TNFSF14* (all signalling via LTβR) and *CD40L* were all spatially associated with FRC-like cells, expressed by B- and T-cells (with LIGHT expressed by FRC-like), potentially driving the FRC-like phenotypic transition. These ligands have all been strongly implicated in TLS neogenesis [43]. Notably, *LTBR* was expressed by all fibroblast subsets (including CAF), suggesting a common capability to respond to LTBR ligands. In vitro, treatment of primary fibroblasts with lymphotoxin induced FRC-like genes (*CCL19*,* CCL21*,* SPIB*) which was enhanced by inhibiting TGFβ signalling.

TLS have been reported in a variety of cancers including HNSCC and NSCLC [44, 45], but their occurrence likely differs between cancer types. This perhaps is reflected in our pan-cancer fibroblast atlas; FRC-like cells were found with highest average relative abundance in HPV+ve HNSCC (11.2%) followed by lung cancer (1.7%). Rarer phenotypes are under-represented in scRNASeq and the distinct FRC-like clusters in HNSCC and pan-cancer analysis was likely aided by inclusion of an FRC-like-rich cancer type (HPV+ve HNSCC). The pan-cancer analysis demonstrated that FRC-like fibroblasts are present in all cancer types but with low abundance, and thus probably do not cluster discretely when datasets are analysed separately. It is also noteworthy that we detected FRC-like fibroblasts in HPV-ve Visium sections, but not using scRNA-Seq. This highlights the power of deriving cell type specific gene signatures from scRNA-Seq data and using this to deconvolute spatial transcriptomic analysis of tissue sections: enabling far greater numbers of cells to be profiled and avoiding the challenges associated with isolating stromal cells from tissue through disaggregation.

The presence of TLS is associated with favourable prognosis in many cancer types, including HNSCC [32, 44], in part reflecting the presence of an ongoing, antigen-dependent immune response [45]. Moreover, the presence of TLS has been shown to predict for response to immunotherapy response in several cancer types [33, 39]. Our analysis of HNSCC, lung cancer and melanoma patients treated with immune checkpoint blockade shows that high levels of FRC-like fibroblasts in tumours are associated with significantly improved survival suggesting that higher levels of FRC-like fibroblasts may identify likely responders. Furthermore, given their central role in TLS organisation and maintenance, generating FRC-like fibroblasts could be an attractive therapeutic strategy to potentiate immunotherapy response.

Recent studies have identified iCAF in several tumour types, including pancreatic cancer and breast cancer [5, 6, 17, 38], using a variety of markers that encompass inflammatory cytokines and other genes (*CXCL1*, *CXCL8*, *CXCL12*, *IL6*, *CFD*, *DPT*) [5]. Using a frequently used iCAF gene signature [5], we identified several fibroblast clusters enriched in the pan-cancer analysis that shared expression of genes such as *CXCL8*, *CXCL1*, *CXCL2*, *IL6*, with some phenotypes present in normal tissue (e.g., *CXCL8* + breast fibroblasts; a similar fibroblast population has been described previously in breast tissue as ‘Fibro-major’ [46]). Of the iCAF signature expressing subsets specific to cancers, *IGF1 +* CAF and *IL11 +* CAF were abundant in tumours. *IGF1 +* CAF were present in all tumour types and expressed all iCAF markers originally identified in PDAC. *IGF1 +* CAF were transcriptionally similar to universal (*PI16+*) fibroblasts, maintaining expression of universal fibroblast genes (*PI16*, *PLA2G2A*, *CFD*) but showed evidence of activation (expression of *FAP*, *COL1A1*, *IGF1*) and expression of iCAF markers (*IL6*, *CXCL8*, *CXCL2*). Within the HNSCC dataset, ~ 50% of PI16 labelled fibroblasts from tumour samples were labelled as *IGF1 +* CAF in the pan-cancer analysis, suggesting that this phenotype is an early/low activation phenotype consistent with previous studies [1, 47]. *IGF1* has been reported to mark iCAF in several cancer types [17, 48], and this low activation subset probably represents the most commonly referenced ‘iCAF’ phenotype in the literature currently.

*IL11 +* CAF expressed significantly higher levels of inflammatory genes compared to *IGF1 +* CAF. These were prevalent in GI tumours (HNSCC, CRC, ESCC), but not detected in breast or lung cancers. Although *IL11 +* CAF could be found with epithelial cells (unlike previous work highlighting iCAF to be distant to epithelial cells; [49]), they especially correlated with inflammatory monocytes and neutrophils. A recent large scRNA-Seq analysis of colorectal tumours revealed a ‘myeloid-cell-attracting’ hub consisting of inflammatory monocytes, neutrophils and *MMP3 +* CAF [50] hypothesised to be associated with tissue damage and microbial products. An association between inflammatory fibroblasts and myeloid cells has also been described in autoimmune inflammatory bowel disease [51] and periodontitis [52], suggesting this inflammatory niche exists beyond cancer.

Gene enrichment and pseudotime analyses identified canonical NF-κB and JAK/STAT signalling as regulating the *IL11 +* CAF phenotype, with IL-1β and TNF-α as likely ligands. Treatment of normal oropharyngeal fibroblasts with IL1β and TNF-α combination upregulated genes expressed by this phenotype in vivo. Consistent with the spatial analysis, treatment of fibroblasts with conditioned media from inflammatory monocytes treatment produced similar results. The immunological role of *IL11 +* CAF in cancer is not clear, but highly expressed genes, including IL-6 cytokine family (*IL6*,* IL11*,* OSM*) are associated with immunotherapy resistance [12]. The *IL11 +* CAF subset was associated with significantly poorer overall survival in immunotherapy-treated HNSCC patients.

## Conclusion

In conclusion, single cell analysis of HNSCC identifies inflammatory fibroblast subsets that are associated with distinct immune cell niches: *CCL19 + *FRC-like with CD4 + T-cells and B-cells; *IL11 +* CAF with inflammatory monocytes and neutrophils. Immune-hot HPV+ve HNSCC contain significantly higher levels of FRC-like fibroblasts; their spatial location within TLS, and their positive association with immunotherapy response suggests that these cells support anti-tumour immunity. We also identify transcriptionally discrete iCAF phenotypes including a low activation/transition phenotype (*IGF1+*), likely the predominant iCAF in the current literature, as well as a more highly inflammatory *IL11 +* CAF subtype found within cancers of the GI tract. Distinguishing between these phenotypes and dissecting functional differences will be important considerations going forwards. It is intriguing that immunological differences within tumours may be tied to fibroblast phenotypes, and the association of fibroblast subtypes with both negative and positive effects on anti-tumour immunity raises intriguing therapeutic possibilities.

## Electronic supplementary material

Below is the link to the electronic supplementary material.

Supplementary Material 1

Supplementary Material 2

Supplementary Material 3

Supplementary Material 4

Supplementary Material 5

Supplementary Material 6

Supplementary Material 7

Supplementary Material 8

Supplementary Material 9

## Data Availability

scRNA-Seq data generated in this study is available at CZ CELLxGENE (https://cellxgene.cziscience.com/collections/3c34e6f1-6827-47dd-8e19-9edcd461893f). All spatial transcriptomics data from this study are available from the Zenodo data repository (10.5281/zenodo.14284038). Seurat objects for HNSCC and PCFA fibroblast integrated scRNA-Seq analyses are available from the Zenodo data repository (10.5281/zenodo.14284357). R scripts used in this study are available on Github (https://github.com/cjh-lab/MolCancer_HNSCCfibs). Raw data generated in this study are available from the corresponding author on reasonable request. The data analysed in this study obtained from GEO: GSE164690, GSE161529, GSE150290, GSE160269, GSE178341, GSE129455, GSE159067 and GSE161537. PRJCA001063 was retrieved from the Genome Sequence Archive. PRJEB23709 was accessed through http://tide.dfci.harvard.edu/download/.

## Abbreviations

CAF: Cancer-associated fibroblast
HNSCC: Head and neck squamous cell carcinoma
HPV: Human papillomavirus
iCAF: Inflammatory CAF
FRC: Fibroblastic reticular cell
ECM: Extracellular matrix
myCAF: Myofibroblastic CAF
apCAF: Antigen-presenting CAF
meCAF: Metabolic CAF
PDAC: Pancreatic ductal adenocarcinoma
TIL: Tumour infiltrating lymphocytes
GI: Gastrointestinal
UMAP: Uniform Manifold Approximation and Projection
DEG: Differentially expressed gene
NSCLC: Non-small cell lung cancer
GSEA: Gene set enrichment analysis
ssGSEA: Single sample gene set enrichment analysis
scRNA-Seq: Single cell RNA-Sequencing
MxIF: Multiplexed immunofluorescence
Tfh: T follicular helper
GC: Geminal centre
TF: Transcription factor
ROI: Region of interest
RCTD: Robust cell type deconvolution
TLS: Tertiary lymphoid structure
LTβR: Lymphotoxin beta receptor
LT: Lymphotoxin α1β2
NOF: Normal primary oral fibroblast
PCFA: Pan-cancer fibroblast atlas
Fib: Fibroblast
CRC: Colorectal carcinoma
ESCC: Oesophageal squamous cell carcinoma
MALT: Mucosa-associated lymphoid tissues
SLO: Secondary lymphoid organ

## References

[1] Buechler MB, Pradhan RN, Krishnamurty AT, Cox C, Calviello AK, Wang AW, et al. Cross-tissue organization of the fibroblast lineage. Nature. 2021;593:575–9. 33981032 10.1038/s41586-021-03549-5

[2] Davidson S, Coles M, Thomas T, Kollias G, Ludewig B, Turley S, et al. Fibroblasts as immune regulators in infection, inflammation and cancer. Nat Rev Immunol. 2021;21:704–17. 33911232 10.1038/s41577-021-00540-z

[3] Hanley CJ, Waise S, Ellis MJ, Lopez MA, Pun WY, Taylor J, et al. Single-cell analysis reveals prognostic fibroblast subpopulations linked to molecular and immunological subtypes of lung cancer. Nat Commun. 2023;14:387. 36720863 10.1038/s41467-023-35832-6 PMC9889778

[4] Biffi G, Oni TE, Spielman B, Hao Y, Elyada E, Park Y, et al. IL1-Induced JAK/STAT signaling is antagonized by TGFβ to shape CAF heterogeneity in pancreatic ductal adenocarcinoma. Cancer Discov. 2019;9:282–301. 30366930 10.1158/2159-8290.CD-18-0710 PMC6368881

[5] Elyada E, Bolisetty M, Laise P, Flynn WF, Courtois ET, Burkhart RA, et al. Cross-species single-cell analysis of pancreatic ductal adenocarcinoma reveals Antigen-Presenting Cancer-Associated fibroblasts. Cancer Discov. 2019;9:1102–23. 31197017 10.1158/2159-8290.CD-19-0094 PMC6727976

[6] Kieffer Y, Hocine HR, Gentric G, Pelon F, Bernard C, Bourachot B, et al. Single-cell analysis reveals fibroblast clusters linked to Immunotherapy Resistance in Cancer. Cancer Discov. 2020;10:1330–51. 32434947 10.1158/2159-8290.CD-19-1384

[7] Lambrechts D, Wauters E, Boeckx B, Aibar S, Nittner D, Burton O, et al. Phenotype molding of stromal cells in the lung tumor microenvironment. Nat Med. 2018;24:1277–89. 29988129 10.1038/s41591-018-0096-5

[8] Krausgruber T, Fortelny N, Fife-Gernedl V, Senekowitsch M, Schuster LC, Lercher A, et al. Structural cells are key regulators of organ-specific immune response. Nature. 2020;583:296–302. 32612232 10.1038/s41586-020-2424-4 PMC7610345

[9] Dammeijer F, van Gulijk M, Mulder EE, Lukkes M, Klaase L, Bosch T, van den, et al. The PD-1/PD-L1-Checkpoint restrains T cell immunity in Tumor-Draining Lymph Nodes. Cancer Cell. 2020;38:685–e7008. 33007259 10.1016/j.ccell.2020.09.001

[10] Ford K, Hanley CJ, Mellone M, Szyndralewiez C, Heitz F, Wiesel P, et al. NOX4 inhibition potentiates immunotherapy by overcoming Cancer-Associated fibroblast-mediated CD8 T-cell exclusion from tumors. Cancer Res. 2020;canres:0008–CAN5472. 10.1158/0008-5472.CAN-19-3158 PMC7611230 32122909

[11] Mariathasan S, Turley SJ, Nickles D, Castiglioni A, Yuen K, Wang Y, et al. TGFβ attenuates tumour response to PD-L1 blockade by contributing to exclusion of T cells. Nature. 2018;554:544–8. 29443960 10.1038/nature25501 PMC6028240

[12] Tsukamoto H, Fujieda K, Miyashita A, Fukushima S, Ikeda T, Kubo Y, et al. Combined blockade of IL6 and PD-1/PD-L1 signaling abrogates mutual regulation of their immunosuppressive effects in the Tumor Microenvironment. Cancer Res. 2018;78:5011–22. 29967259 10.1158/0008-5472.CAN-18-0118

[13] Croft AP, Campos J, Jansen K, Turner JD, Marshall J, Attar M, et al. Distinct fibroblast subsets drive inflammation and damage in arthritis. Nature. 2019;570:246–51. 31142839 10.1038/s41586-019-1263-7 PMC6690841

[14] Grauel AL, Nguyen B, Ruddy D, Laszewski T, Schwartz S, Chang J, et al. TGFβ-blockade uncovers stromal plasticity in tumors by revealing the existence of a subset of interferon-licensed fibroblasts. Nat Commun. 2020;11:6315. 33298926 10.1038/s41467-020-19920-5 PMC7725805

[15] Galon J, Bruni D. Approaches to treat immune hot, altered and cold tumours with combination immunotherapies. Nat Rev Drug Discov. 2019;18:197–218. 30610226 10.1038/s41573-018-0007-y

[16] Ward MJ, Thirdborough SM, Mellows T, Riley C, Harris S, Suchak K, et al. Tumour-infiltrating lymphocytes predict for outcome in HPV-positive oropharyngeal cancer. Br J Cancer. 2014;110:489–500. 24169344 10.1038/bjc.2013.639 PMC3899750

[17] Cords L, Tietscher S, Anzeneder T, Langwieder C, Rees M, de Souza N, et al. Cancer-associated fibroblast classification in single-cell and spatial proteomics data. Nat Commun. 2023;14:4294. 37463917 10.1038/s41467-023-39762-1 PMC10354071

[18] Dominguez CX, Müller S, Keerthivasan S, Koeppen H, Hung J, Gierke S, et al. Single-cell RNA sequencing reveals stromal evolution into LRRC15 + myofibroblasts as a determinant of patient response to Cancer Immunotherapy. Cancer Discov. 2020;10:232–53. 31699795 10.1158/2159-8290.CD-19-0644

[19] Kuleshov MV, Jones MR, Rouillard AD, Fernandez NF, Duan Q, Wang Z, et al. Enrichr: a comprehensive gene set enrichment analysis web server 2016 update. Nucleic Acids Res. 2016;44:W90–7. 27141961 10.1093/nar/gkw377 PMC4987924

[20] Wu T, Hu E, Xu S, Chen M, Guo P, Dai Z, et al. clusterProfiler 4.0: a universal enrichment tool for interpreting omics data. Innov (Camb). 2021;2:100141. 10.1016/j.xinn.2021.100141 PMC8454663 34557778

[21] Schubert M, Klinger B, Klünemann M, Sieber A, Uhlitz F, Sauer S, et al. Perturbation-response genes reveal signaling footprints in cancer gene expression. Nat Commun. 2018;9:20. 29295995 10.1038/s41467-017-02391-6 PMC5750219

[22] Dann E, Henderson NC, Teichmann SA, Morgan MD, Marioni JC. Differential abundance testing on single-cell data using k-nearest neighbor graphs. Nat Biotechnol. 2022;40:245–53. 34594043 10.1038/s41587-021-01033-z PMC7617075

[23] Garcia-Alonso L, Holland CH, Ibrahim MM, Turei D, Saez-Rodriguez J. Benchmark and integration of resources for the estimation of human transcription factor activities. Genome Res. 2019;29:1363–75. 31340985 10.1101/gr.240663.118 PMC6673718

[24] Cerami E, Gao J, Dogrusoz U, Gross BE, Sumer SO, Aksoy BA, et al. The cBio cancer genomics portal: an open platform for exploring multidimensional cancer genomics data. Cancer Discov. 2012;2:401–4. 22588877 10.1158/2159-8290.CD-12-0095 PMC3956037

[25] Becht E, Giraldo NA, Lacroix L, Buttard B, Elarouci N, Petitprez F, et al. Estimating the population abundance of tissue-infiltrating immune and stromal cell populations using gene expression. Genome Biol. 2016;17:218. 27765066 10.1186/s13059-016-1070-5 PMC5073889

[26] Kürten CHL, Kulkarni A, Cillo AR, Santos PM, Roble AK, Onkar S, et al. Investigating immune and non-immune cell interactions in head and neck tumors by single-cell RNA sequencing. Nat Commun. 2021;12:7338. 34921143 10.1038/s41467-021-27619-4 PMC8683505

[27] Hynes RO, Naba A. Overview of the matrisome–an inventory of extracellular matrix constituents and functions. Cold Spring Harb Perspect Biol. 2012;4:a004903. 21937732 10.1101/cshperspect.a004903 PMC3249625

[28] Bonizzi G, Karin M. The two NF-κB activation pathways and their role in innate and adaptive immunity. Trends Immunol. 2004;25:280–8. 15145317 10.1016/j.it.2004.03.008

[29] Cable DM, Murray E, Zou LS, Goeva A, Macosko EZ, Chen F, et al. Robust decomposition of cell type mixtures in spatial transcriptomics. Nat Biotechnol. 2022;40:517–26. 33603203 10.1038/s41587-021-00830-w PMC8606190

[30] Dong R, Yuan G-C. SpatialDWLS: accurate deconvolution of spatial transcriptomic data. Genome Biol. 2021;22:145. 33971932 10.1186/s13059-021-02362-7 PMC8108367

[31] Fletcher AL, Acton SE, Knoblich K. Lymph node fibroblastic reticular cells in health and disease. Nat Rev Immunol. 2015;15:350–61. 25998961 10.1038/nri3846 PMC5152733

[32] Sautès-Fridman C, Petitprez F, Calderaro J, Fridman WH. Tertiary lymphoid structures in the era of cancer immunotherapy. Nat Rev Cancer. 2019;19:307–25. 31092904 10.1038/s41568-019-0144-6

[33] Cabrita R, Lauss M, Sanna A, Donia M, Skaarup Larsen M, Mitra S, et al. Tertiary lymphoid structures improve immunotherapy and survival in melanoma. Nature. 2020;577:561–5. 31942071 10.1038/s41586-019-1914-8

[34] Coppola D, Nebozhyn M, Khalil F, Dai H, Yeatman T, Loboda A, et al. Unique ectopic lymph node-like structures present in human primary colorectal carcinoma are identified by immune gene array profiling. Am J Pathol. 2011;179:37–45. 21703392 10.1016/j.ajpath.2011.03.007 PMC3123872

[35] Gu-Trantien C, Loi S, Garaud S, Equeter C, Libin M, de Wind A, et al. CD4 + follicular helper T cell infiltration predicts breast cancer survival. J Clin Invest. 2013;123:2873–92. 23778140 10.1172/JCI67428 PMC3696556

[36] Hennequin A, Derangère V, Boidot R, Apetoh L, Vincent J, Orry D, et al. Tumor infiltration by tbet + effector T cells and CD20 + B cells is associated with survival in gastric cancer patients. Oncoimmunology. 2016;5:e1054598. 27057426 10.1080/2162402X.2015.1054598 PMC4801425

[37] Browaeys R, Saelens W, Saeys Y. NicheNet: modeling intercellular communication by linking ligands to target genes. Nat Methods. 2020;17:159–62. 31819264 10.1038/s41592-019-0667-5

[38] Ma C, Yang C, Peng A, Sun T, Ji X, Mi J, et al. Pan-cancer spatially resolved single-cell analysis reveals the crosstalk between cancer-associated fibroblasts and tumor microenvironment. Mol Cancer. 2023;22:170. 37833788 10.1186/s12943-023-01876-x PMC10571470

[39] Helmink BA, Reddy SM, Gao J, Zhang S, Basar R, Thakur R, et al. B cells and tertiary lymphoid structures promote immunotherapy response. Nature. 2020;577:549–55. 31942075 10.1038/s41586-019-1922-8 PMC8762581

[40] Puram SV, Tirosh I, Parikh AS, Patel AP, Yizhak K, Gillespie S, et al. Single-cell transcriptomic analysis of primary and metastatic Tumor ecosystems in Head and Neck Cancer. Cell. 2017;171:1611–e162424. 29198524 10.1016/j.cell.2017.10.044 PMC5878932

[41] Nayar S, Campos J, Smith CG, Iannizzotto V, Gardner DH, Mourcin F, et al. Immunofibroblasts are pivotal drivers of tertiary lymphoid structure formation and local pathology. Proc Natl Acad Sci USA. 2019;116:13490–7. 31213547 10.1073/pnas.1905301116 PMC6613169

[42] Remouchamps C, Boutaffala L, Ganeff C, Dejardin E. Biology and signal transduction pathways of the Lymphotoxin-αβ/LTβR system. Cytokine Growth Factor Rev. 2011;22:301–10. 22152226 10.1016/j.cytogfr.2011.11.007

[43] Kang W, Feng Z, Luo J, He Z, Liu J, Wu J, et al. Tertiary lymphoid structures in Cancer: the double-edged Sword Role in Antitumor Immunity and potential therapeutic induction strategies. Front Immunol. 2021;12:689270. 34394083 10.3389/fimmu.2021.689270 PMC8358404

[44] Ruffin AT, Cillo AR, Tabib T, Liu A, Onkar S, Kunning SR, et al. B cell signatures and tertiary lymphoid structures contribute to outcome in head and neck squamous cell carcinoma. Nat Commun. 2021;12:3349. 34099645 10.1038/s41467-021-23355-x PMC8184766

[45] Schumacher TN, Thommen DS. Tertiary lymphoid structures in cancer. Science. 2022;375:eabf9419. 34990248 10.1126/science.abf9419

[46] Kumar T, Nee K, Wei R, He S, Nguyen QH, Bai S, et al. A spatially resolved single-cell genomic atlas of the adult human breast. Nature. 2023;620:181–91. 37380767 10.1038/s41586-023-06252-9 PMC11443819

[47] Grout JA, Sirven P, Leader AM, Maskey S, Hector E, Puisieux I, et al. Spatial positioning and matrix programs of cancer-associated fibroblasts promote T cell exclusion in human lung tumors. Cancer Discov. 2022;12:2606–25. 36027053 10.1158/2159-8290.CD-21-1714 PMC9633420

[48] Chen Z, Zhou L, Liu L, Hou Y, Xiong M, Yang Y, et al. Single-cell RNA sequencing highlights the role of inflammatory cancer-associated fibroblasts in bladder urothelial carcinoma. Nat Commun. 2020;11:5077. 33033240 10.1038/s41467-020-18916-5 PMC7545162

[49] Öhlund D, Handly-Santana A, Biffi G, Elyada E, Almeida AS, Ponz-Sarvise M, et al. Distinct populations of inflammatory fibroblasts and myofibroblasts in pancreatic cancer. J Exp Med. 2017;214:579–96. 28232471 10.1084/jem.20162024 PMC5339682

[50] Pelka K, Hofree M, Chen JH, Sarkizova S, Pirl JD, Jorgji V, et al. Spatially organized multicellular immune hubs in human colorectal cancer. Cell. 2021;184:4734–e475220. 34450029 10.1016/j.cell.2021.08.003 PMC8772395

[51] Smillie CS, Biton M, Ordovas-Montañes J, Sullivan KM, Burgin G, Graham DB, et al. Cellular and inter-cellular rewiring of the human colon during ulcerative colitis. Cell. 2019;178:714–e73022. 31348891 10.1016/j.cell.2019.06.029 PMC6662628

[52] Williams DW, Greenwell-Wild T, Brenchley L, Dutzan N, Overmiller A, Sawaya AP, et al. Human oral mucosa cell atlas reveals a stromal-neutrophil axis regulating tissue immunity. Cell. 2021;184:4090–e410415. 34129837 10.1016/j.cell.2021.05.013 PMC8359928

