# Supplementary material for: Single cell and spatial analysis of immune-hot and immune-cold tumours identifies fibroblast subtypes associated with distinct immunological niches and positive immunotherapy response
Source: Mol Cancer. 2025 Jan 6;24:3. doi: 10.1186/s12943-024-02191-9 (PMC11702232; doi:10.1186/s12943-024-02191-9)
Supplement: Supplementary file 1 — Supplementary Material 1 [file 12943_2024_2191_MOESM1_ESM.docx]

**SUPPLEMENTARY MATERIALS**

**Single cell and spatial analysis of immune-hot and immune-cold tumours identifies fibroblast subtypes associated with distinct immunological niches and positive immunotherapy response**

Benjamin H Jenkins^1,2^, Ian Tracy^1,2^, Maria Fernanda SD Rodrigues^1,2,3^, Melanie JL Smith^1,2^, Begoña R Martinez^1,2^, Mark Edmond^1,4^, Sangeetha Mahadevan^5^, Anjali Rao^5^, Hailing Zong^5^, Kai Liu^5^, Abhishek Aggarwal^5^, Li Li,^5^ Lauri Diehl^5^, Emma V King^1,4^, Jamie G Bates^5*^, Christopher J Hanley^1,2*^, Gareth J Thomas^1,2*†^

^1^School of Cancer Sciences, University of Southampton, Southampton, UK

^2^NIHR Experimental Cancer Medicine Centre, University of Southampton, Southampton, UK

^3^Postgraduate program in Medicine-Biophotonics, Nove de Julho University, São Paulo, Brazil

^4^Dorset Cancer Centre, Poole Hospital NHS Foundation Trust, Poole, UK.

^5^Gilead Sciences Inc. Foster City, California, US.

*These authors jointly supervised this work

†Corresponding author

Gareth Thomas, Cancer Sciences Unit, Faculty of Medicine, University of Southampton, Tremona Road

Southampton SO16 65YD

e-mail: [g.thomas@soton.ac.uk](mailto:g.thomas@soton.ac.uk)

**Supplementary Materials:**

# Supplementary Figures

1. Supplementary Materials and Methods

# **1. SUPPLEMENTARY FIGURES**


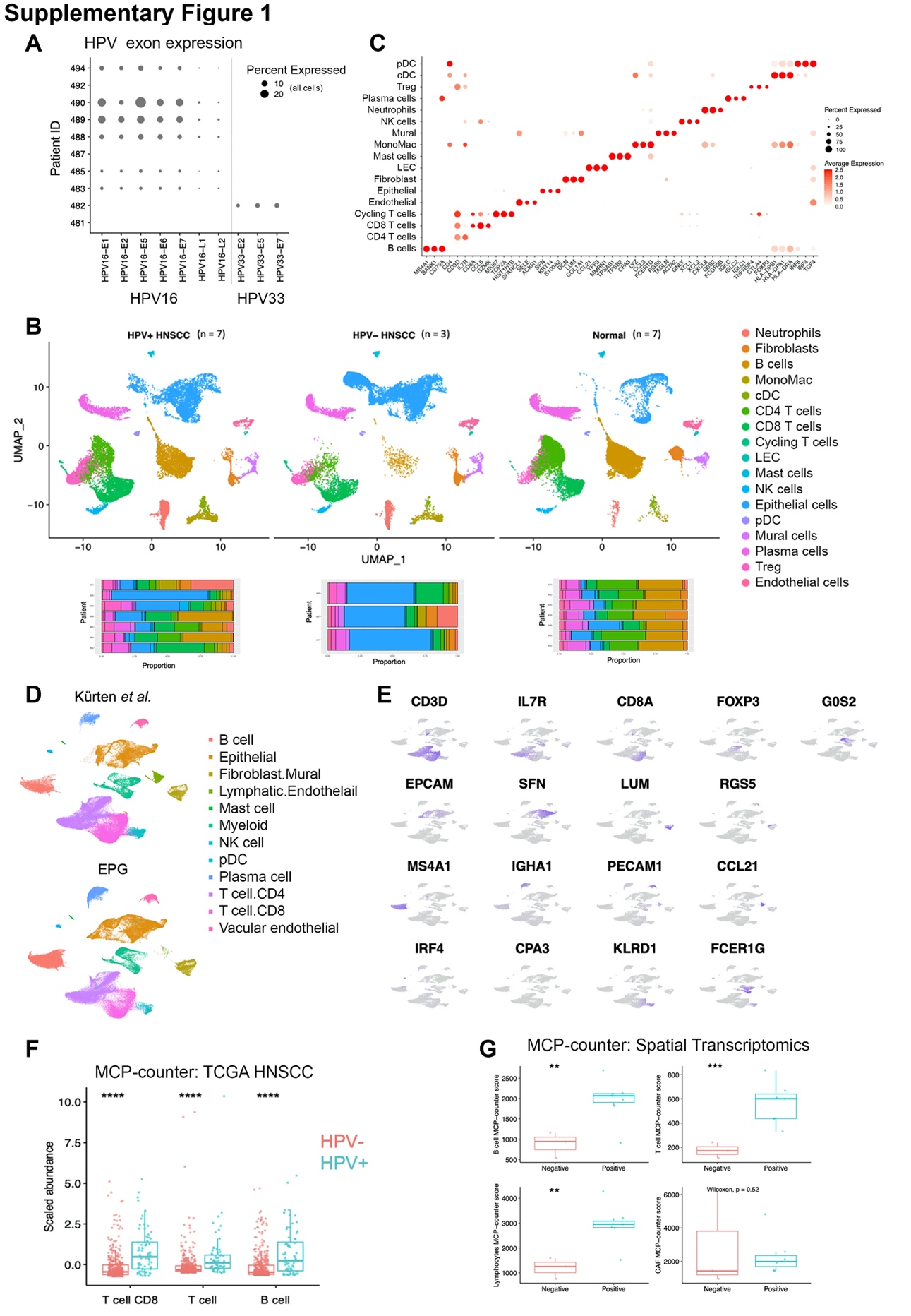


# **Supplementary Figure 1 (related to Figure 1).**

# A) HPV exon expression across all cells in the in-house EPG scRNA-Seq data (n=10). Exons with detected expression are displayed. Samples were sequentially aligned to human reference genome (GRCh38–2020-A) with HPV-16 reference sequence (NC_001526) or HPV-33 (OQ_672679). B) Plot showing UMAP embeddings for integrated (Seurat RPCA) in-house HNSCC scRNA-Seq dataset comprising of HPV+ve HNSCC (n=7), HPV-ve HNSCC (n=3) and normal oropharyngeal tissue (n=7). UMAP plots displaying 17 clusters are accompanied with bar plots showing relative proportions of broad cell types per patient sample. Clusters are annotated based on expression of marker genes as shown in Supplementary Figure 1C. C) Dot plot showing expression of key marker genes of clusters. D) Integrated scRNASeq HNSCC dataset (Figure 1) split by dataset showing cell embeddings from EPG and Kürten et al. datasets. E) Feature plots showing expression of key cell type markers in clusters of the integrated HNSCC scRNA-Seq dataset. F) Deconvolution of TCGA HNSC bulk RNA Seq data using MCP-counter. Scaled abundance imputed by MCP-counter for CD8 T cells, T cells and B cells shown in HPV+ve and HPV-ve patients. Wilcoxon rank-sum test (two-sided). G) MCP-counter deconvoluted (spatial transcriptomics; 10x Visium) summative values for B cells, T cells, total lymphocytes and CAF (total values) per patient in HPV-ve/+ve samples. Students t-test or Wilcoxon rank-sum test (two-sided). *p < 0.05; **p < 0.01; ***p < 0.001. ****p < 0.0001.

**
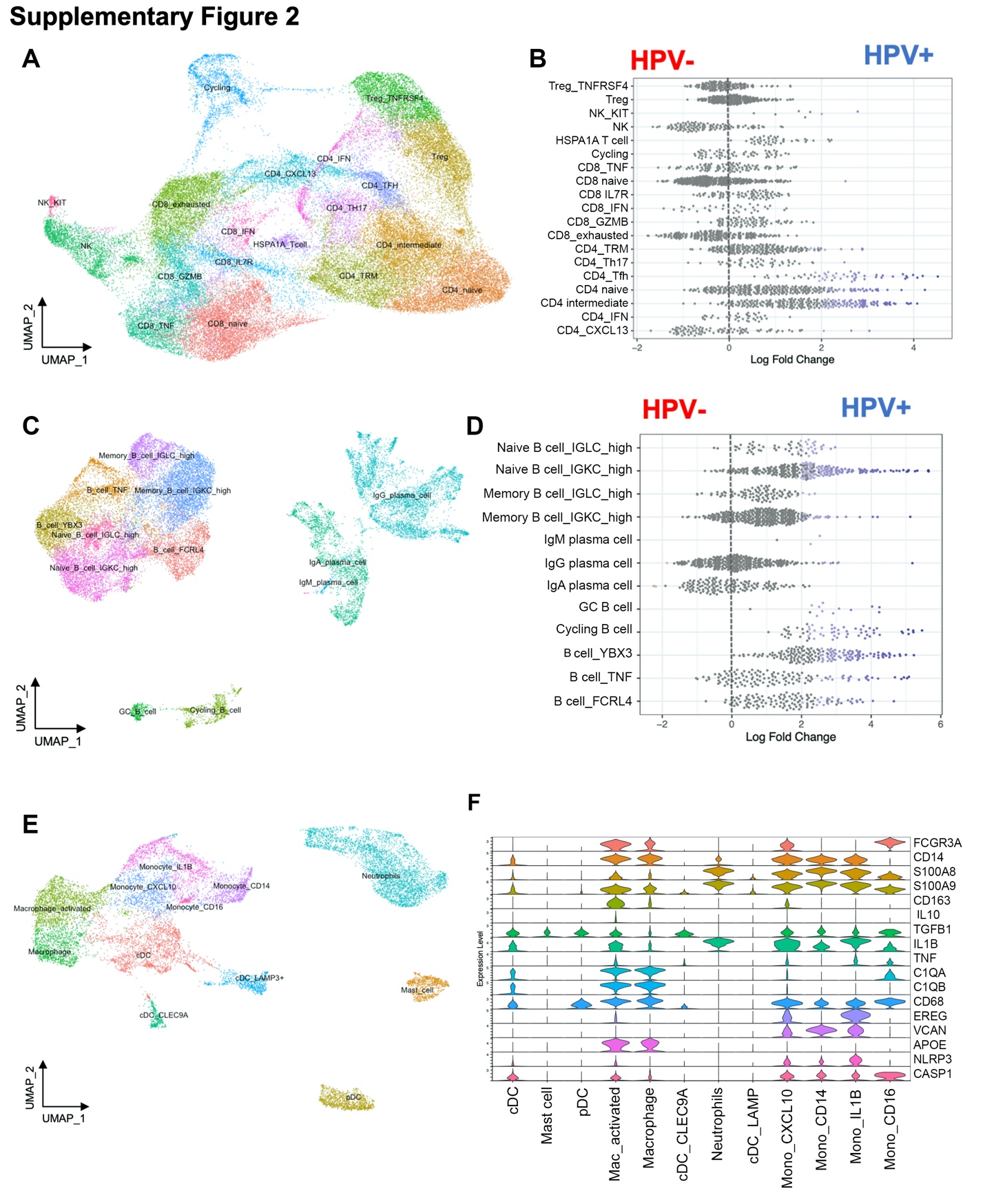
**

**Supplementary Figure 2. UMAP plots of immune cell clusters within integrated HNSCC scRNA-Seq dataset including differential abundance testing in HPV+ve vs HPV-ve samples.**

Immune cell clusters were subset from the original integrated dataset and re-integrated using Seurat RPCA. T/NK cells, B/plasma cells and myeloid cell UMAP plots are shown with annotated clusters. Accompanying these are bee-swarm plots presenting differential abundance testing results between HPV+ve and HPV-ve samples. A) Integrated HNSCC scRNASeq UMAP of T and NK cells. B) T/NK cell differential abundance using MiloR. C) Integrated HNSCC scRNASeq UMAP of B/plasma cells. D) B/plasma cell differential abundance using MiloR. E) Integrated HNSCC scRNASeq UMAP of myeloid cells (which had no differentially abundant clusters between HPV+ve and HPV-ve tumours). F) Selected marker gene expression across HNSCC myeloid cell clusters.


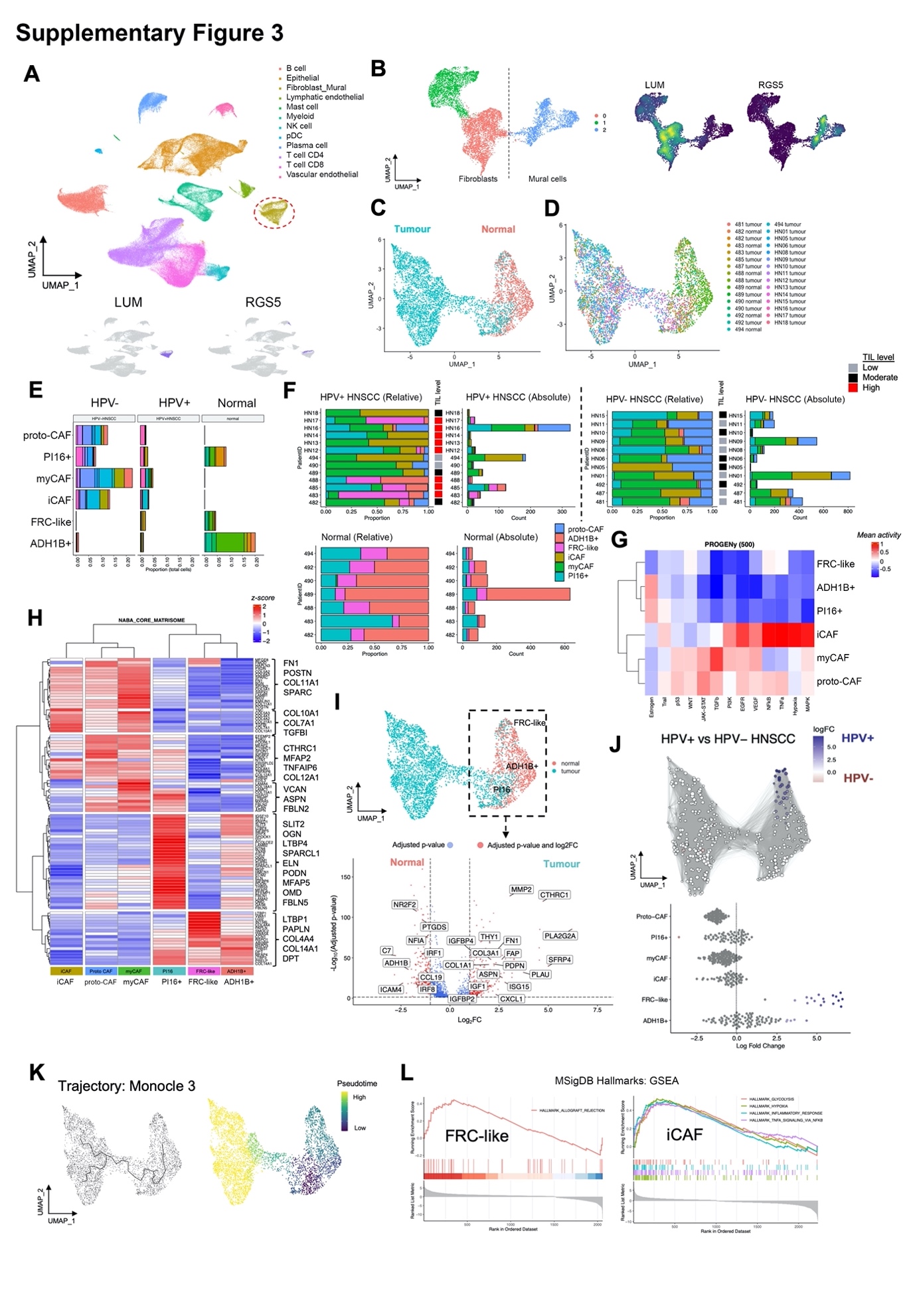


**Supplementary Figure 3. HNSCC fibroblast identification and abundance (related to Figure 2).**

A) Integrated HNSCC dataset UMAP with LUM+ fibroblasts and RGS5+ mural cells highlighted (in the Fibroblast.Mural cluster). B) UMAP plot showing close fibroblast-mural transcriptomic relationship. Three clusters emerged, with two clusters of *LUM+RGS5*- fibroblasts and one cluster of *RGS5+LUM*- mural cells. C) Fibroblast UMAP grouped by source of material: normal or tumour. D) Fibroblast UMAP grouped by patient sample. E) Abundance of each fibroblast cluster as a proportion of total cells per sample. Bar plot coloured by patient sample (panel D). F) Fibroblast bar plots showing relative abundance (proportion of total fibroblasts), and cell counts of fibroblast subsets per patient, split by normal, HPV+ HNSCC and HPV- HNSCC. Sample TIL levels are labelled High (red), Moderate (black) and Low (grey). G) Heatmap showing summarised (mean) activity scores of fibroblasts for pathway responsive genes using PROGENy. H) Average expression of core matrisome [1] genes. Differentially expressed genes (DEGs) identified between all fibroblast clusters (using FindAllMarkers) were filtered for core matrisome genes. I) Volcano plot of DEGs between normal and tumour samples in normal fibroblast phenotypes. PI16/ADH1B+/FRC-like clusters (marked by box on UMAP plot) were subset and FindMarkers used to identify DEGs between normal and tumour samples. Selected genes (log2FC >1; adjusted pval<0.05) are displayed. J) Differential abundance testing between HPV+ve and HPV-ve HNSCC. Highlighting differentially abundant neighbourhoods. K) Single cell trajectory analysis using Monocle 3. UMAPs show overlay of determined trajectory graph path and corresponding pseudotime values. L) Gene set enrichment analysis showing significant (p<0.05) MSigDB Hallmarks gene sets enrichment in FRC-like and iCAF.


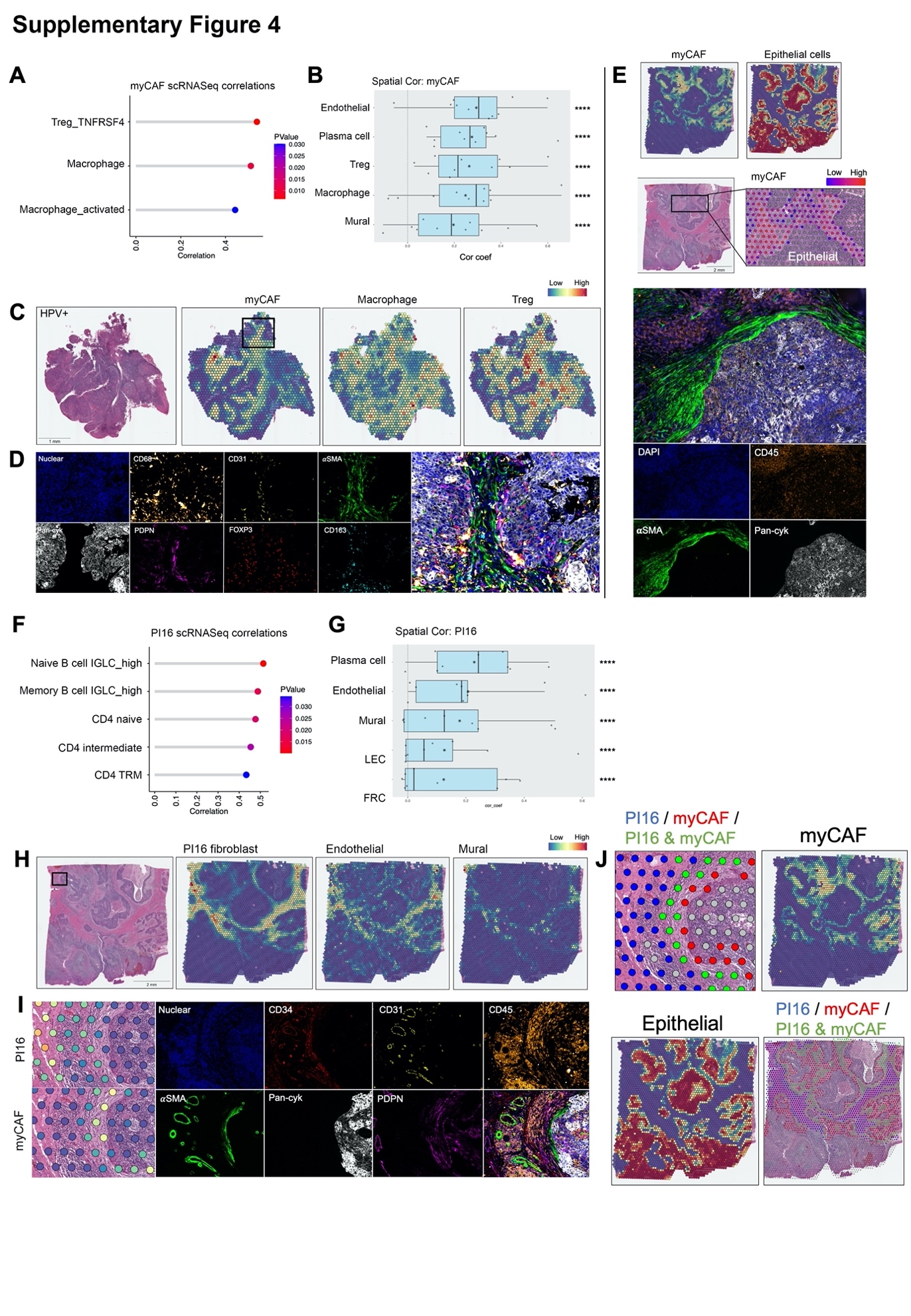


**Supplementary Figure 4. myCAF and PI16+ fibroblasts.**

A) myCAF and immune cell sample-level scRNA-Seq correlations (spearman; p<0.05). For HNSCC samples only, fibroblast proportions (relative to total fibroblasts) per sample were correlated against immune cell cluster proportions (relative to total immune cells). Only significant positive associations are shown. B) Spatial transcriptomics cell type correlations (spearman) using RCTD imputed abundance (normalised weights ≥ 0.05). Visium (10x) spots were deconvoluted using RCTD. Annotated scRNA-Seq data was used as a reference for derivation of cell-type specific gene signatures that were used to deconvolute the cell types present within each 55μm spot. Spearman correlation of normalised weights was carried out on each patient separately. Correlation coefficients are plotted for each of 10 patients, median displayed as vertical line in boxplot and mean as star symbol. Weighted Fisher’s method was used to combine p values. C) Spatial feature plot of deconvoluted values of myCAF, macrophage and Treg in a HPV+ve HNSCC sample. D) MxIF (Phenocycler-Fusion) showing staining (DAPI, Pan-cytokeratin, PDPN, CD31, αSMA, CD68, CD163, FOXP3) in myCAF containing region of interest identified by RCTD deconvolution. MxIF markers are shown separately and accompanied by composite image of all markers. E) Spatial feature plots (spatial transcriptomics) and MxIF demonstrating myCAF spatial association with the epithelial-stromal border. MxIF staining showing DAPI, CD45, αSMA and Pan-cytokeratin. F) PI16+ universal fibroblasts and immune cell sample-level scRNA-Seq correlations (spearman; p<0.05). For HNSCC samples only, fibroblast proportions (relative to total fibroblasts) per sample were correlated against immune cell cluster proportions (relative to total immune cells). Only significant positive associations are shown. G) Spatial transcriptomics cell type correlations (spearman) using RCTD imputed abundance (normalised weights ≥ 0.05). Visium (10x) spots were deconvoluted using RCTD. Annotated scRNA-Seq data was used as a reference for derivation of cell-type specific gene signatures that were used to deconvolute the cell types present within each 55μm spot. Spearman correlation of normalised weights was carried out on each patient separately. Correlation coefficients are plotted for each of 10 patients, median displayed as vertical line in boxplot and mean as star symbol. Weighted Fisher’s method was used to combine p values. H) Spatial feature plot of deconvoluted values of PI16+ universal fibroblasts, endothelial cells and mural cells in a HPV+ve HNSCC sample. I) Spatial feature plot and MxIF (Phenocycler-Fusion) showing staining (DAPI, Pan-cytokeratin, PDPN, CD31, αSMA, CD34, CD45) in PI16+ universal fibroblast containing region of interest identified by RCTD deconvolution. MxIF markers are shown separately and accompanied by composite image of all markers. PI16+ universal fibroblasts express CD34 (CD31-) and are found near vasculature. PDPN and αSMA expression increases and CD34 decreases in fibroblastic cells as distance to epithelial cells (pan-cyk) decreases. This is in parallel to PI16+ universal fibroblast and myCAF spatial localisation according to spatial transcriptomics. J) Spatial feature plots (spatial transcriptomics) demonstrating PI16+ universal fibroblast distanced from epithelial-stromal border. In contrast to myCAF found at the epithelial-stromal border. Fibroblast phenotypes considered present in a Visium spot when RCTD normalised weights ≥ 0.05. Spots coloured by fibroblast phenotype (PI16, blue; myCAF, red; PI16 and myCAF; green). Spots considered mixed when within a given spot >1 fibroblast phenotype was present. *p < 0.05; **p < 0.01; ***p < 0.001. ****p < 0.0001.

# **
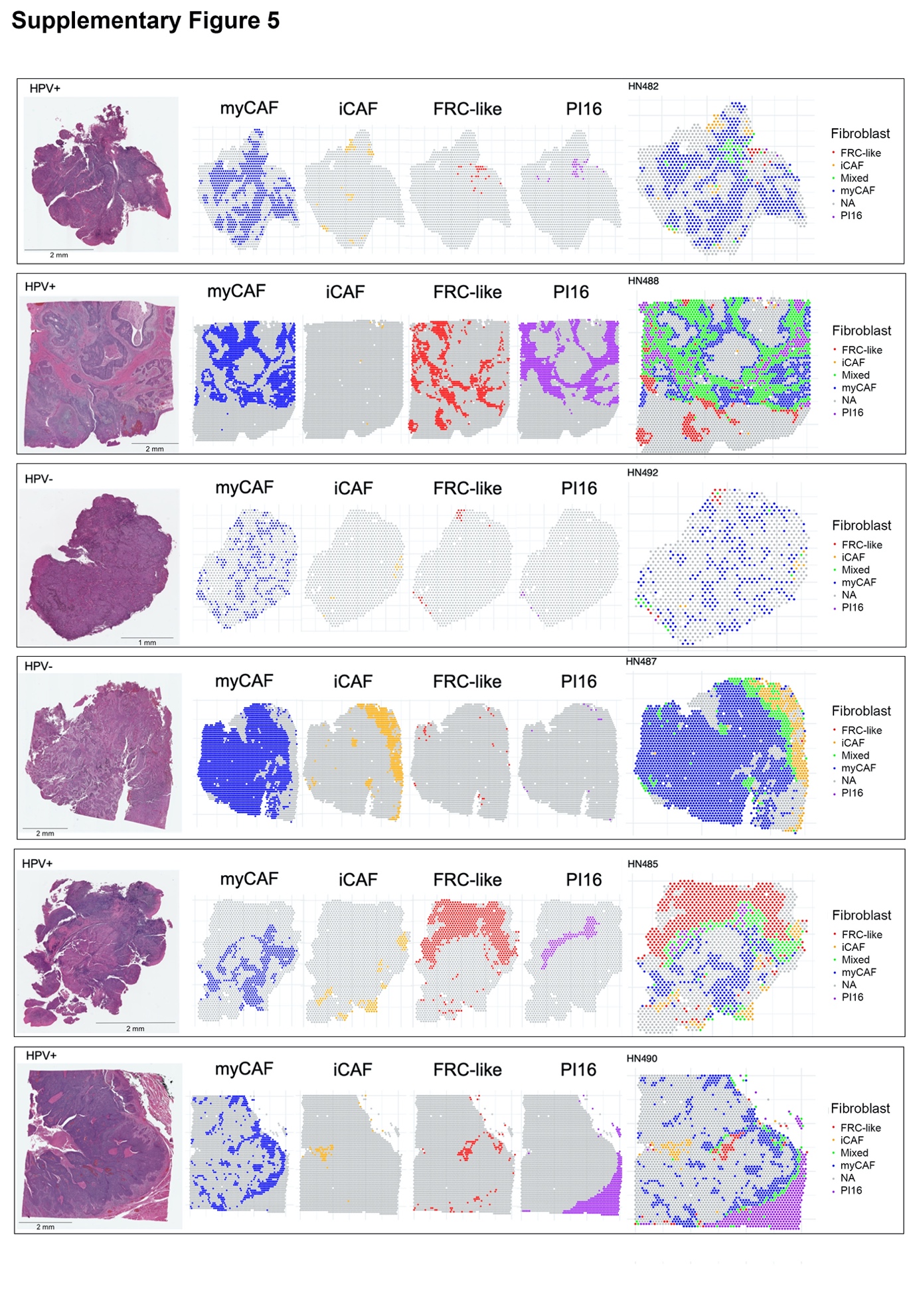
**

**Supplementary Figure 5. Spatial transcriptomics RCTD deconvolution shows fibroblast phenotypes occupy spatially discrete regions across HNSCC sections.**

Fibroblast phenotypes considered present in a Visium spot when RCTD normalised weights ≥ 0.05. Spots coloured by fibroblast phenotype (myCAF, blue; iCAF, orange; FRC-like, red; PI16, purple). Spots considered mixed when within a given spot >1 fibroblast phenotype was present. HPV status depicted above H&E images on the left.

**
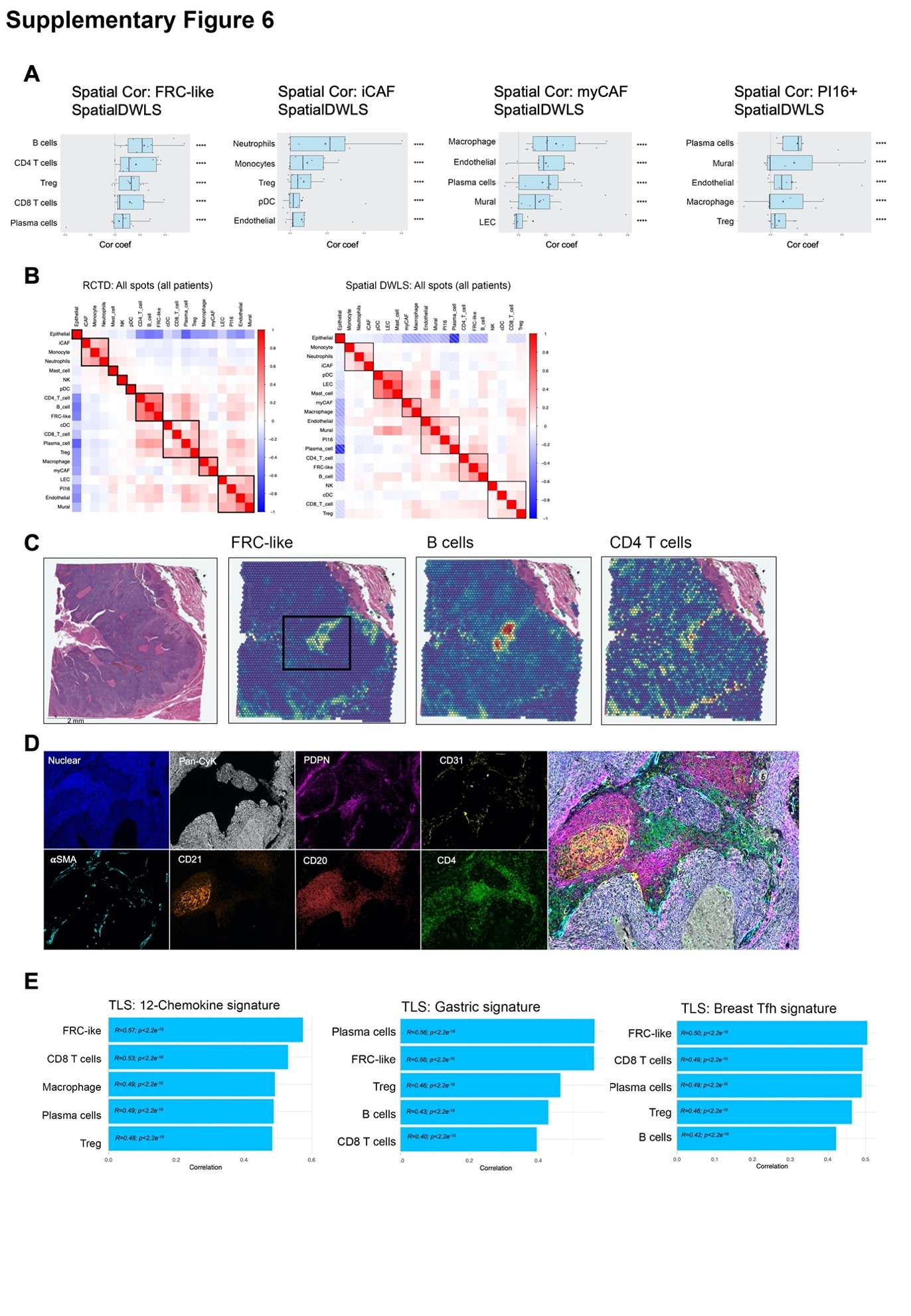
**

**Supplementary Figure 6. Spatial transcriptomics deconvolution cell type correlations using RCTD/SpatialDWLS and FRC-like spatial associations (relating to Figure 3-4).**

A) Spatial transcriptomics cell type correlations (spearman) using SpatialDWLS. Visium (10x) spots were deconvoluted using SpatialDWLS. Annotated scRNA-Seq data was used as a reference for derivation of cell-type specific gene signatures that were used to deconvolute the cell types present within each 55μm spot. Spearman correlation of imputed abundance was carried out on each patient separately. Correlation coefficients are plotted for each of 10 patients, median displayed as vertical line in boxplot and mean as star symbol. Weighted Fisher’s method was used to combine p values. B) Heatmaps of spearman correlation values from spatial analysis using RCTD (normalised weights ≥ 0.05) and SpatialDWLS deconvolution methods. Non-significant associations left blank. Correlations across all spots and all patients. C) Spatial feature plot of deconvoluted values of FRC-like fibroblasts, B cells and CD4+ T cells in a HPV+ve HNSCC sample. D) MxIF (Phenocycler-Fusion) showing staining (DAPI, Pan-cytokeratin, PDPN, CD31, αSMA, CD21, CD20, CD4) in FRC-like containing region of interest identified by RCTD deconvolution. MxIF markers are shown separately and accompanied by composite image of all markers. PDPN+CD31- cells marking fibroblasts. CD21 (CR2) marking follicular dendritic cells found with dense aggregates of B cells (CD20) and CD4+ T cells (CD4). E) FRC-like abundance (RCTD) correlated with module scores for 12-chemokine [2] TLS signature, gastric cancer [3] TLS signature and breast cancer [4] TLS signature. Correlations for each Visium (10x) spot across all 10 patients; top 5 shown. *p < 0.05; **p < 0.01; ***p < 0.001. ****p < 0.0001.

**
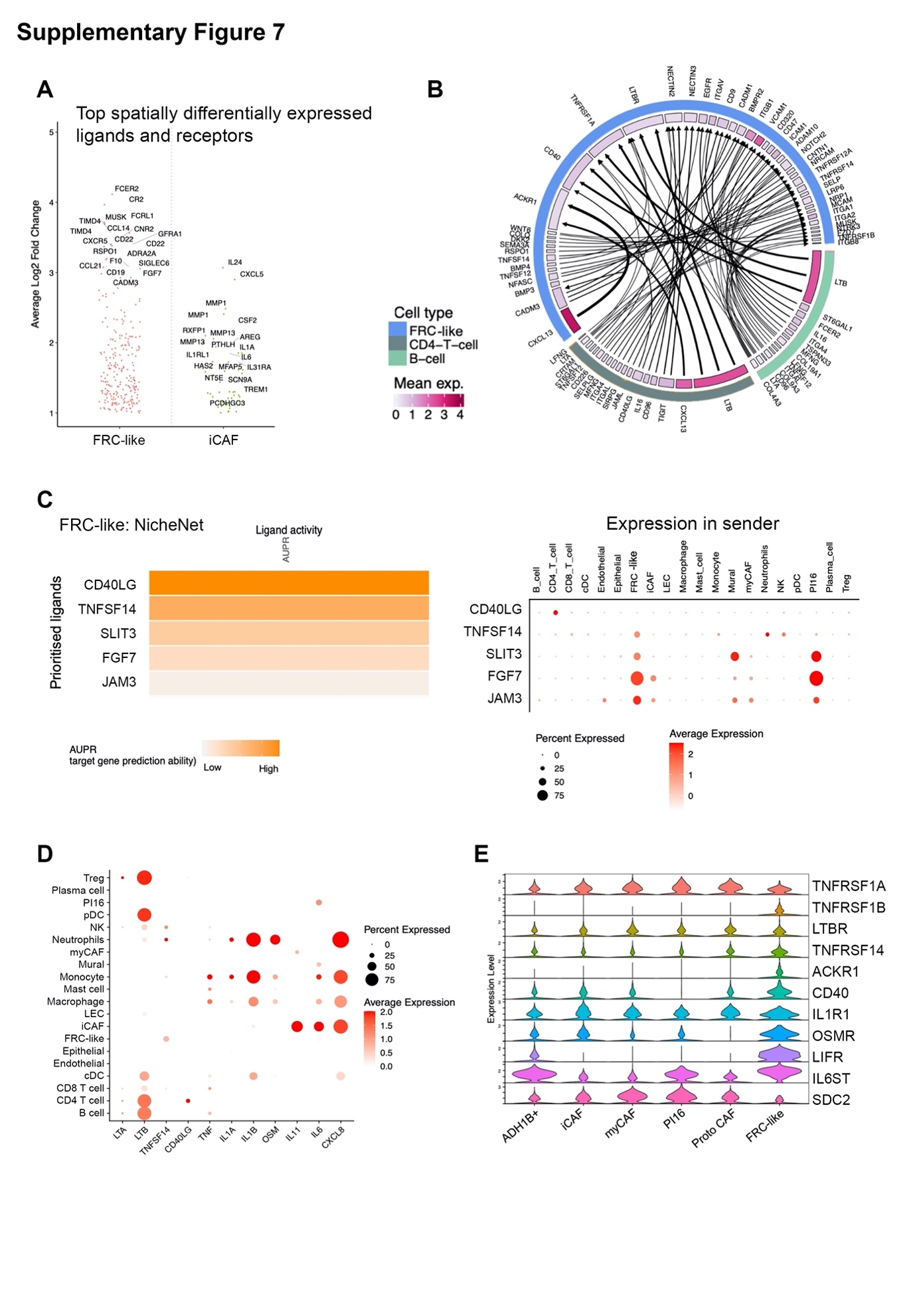
**

**Supplementary Figure 7. Relating to Figure 3.**

A) Differentially expressed (log2FC ≥ 1; padj < 0.0001) ligands and receptors associated with FRC-like and iCAF niche acquired using RCTD depicted spot identification. Differentially expressed ligands identified using FindMarkers on FRC-like/iCAF containing spots (normalised weight ≥ 0.05) filtered for ligands/receptors. Top 10 ligands and top 10 receptors shown. B) Circos plot of all spatially differentially expressed ligands expressed by FRC-like fibroblasts, CD4+ T cells or B cells; and have expression of corresponding receptor in FRC-like fibroblasts. Average normalised expression also shown for each ligand/receptor. C) NicheNet output for top ligands with high target gene prediction ability for FRC-like fibroblast genes. Ligands for analysis were filtered to only contain spatially differentially expressed ligands that had corresponding receptor expression in FRC-like fibroblast. To right, expression of ligands prioritised by NicheNet across the broad cell types in HNSCC scRNA-Seq dataset. D) Expression of selected ligands across the broad cell types in HNSCC scRNA-Seq dataset. E) Expression across fibroblasts of key receptors implicated by ligand-receptor analysis. Demonstrating conserved expression of receptors across phenotypes.

**
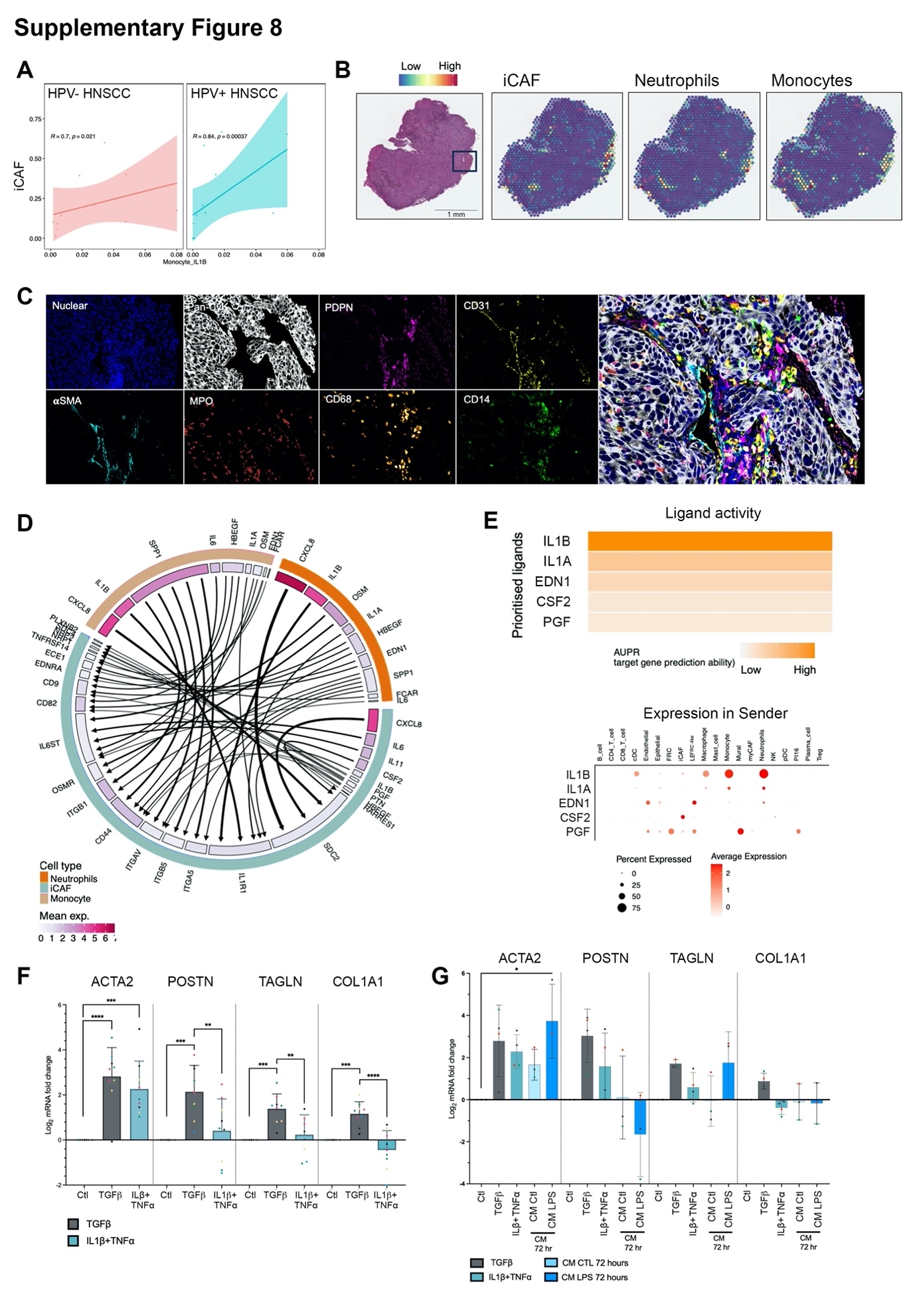
**

**Supplementary Figure 8. iCAF spatial associations (relating to Figure 4).**

A) Sample-level scRNA-Seq correlation (spearman) of iCAF with IL1B inflammatory monocytes. For HNSCC samples only, iCAF proportion (relative to total fibroblasts) per sample were correlated against IL1B monocyte cluster proportions (relative to total immune cells). Graph split by HPV status. B) Spatial feature plot of deconvoluted values of iCAF, monocytes and neutrophils in a HPV-ve HNSCC sample. C) MxIF (Phenocycler-Fusion) showing staining (DAPI, Pan-cytokeratin, PDPN, CD31, αSMA, MPO, CD68, CD14) in iCAF containing region of interest identified by RCTD deconvolution. MxIF markers are shown separately and accompanied by composite image of all markers. D) Circos plot of all spatially differentially expressed ligands expressed by iCAF, monocytes or neutrophils; and have expression of corresponding receptor in iCAF. Average normalised expression also shown for each ligand/receptor. E) NicheNet output for top ligands with high target gene prediction ability for iCAF genes. Ligands for analysis were filtered to only contain spatially differentially expressed ligands that had corresponding receptor expression in iCAF. To right, expression of ligands prioritised by NicheNet across the broad cell types in HNSCC scRNA-Seq dataset. F) qPCR analysis of myCAF markers (ACTA2, POSTN, TAGLN and COL1A1) in n=5 primary NOF lines treated with TGFβ (4ng/mL) or IL1β (1ng/mL)+TNF𝛼 (1ng/mL) for 72 hours. Results show mean ± SD of n=9 independent experiments, colours of points correspond to primary NOF line. One-way ANOVA with Bonferroni correction. G) qPCR analysis of myCAF markers (ACTA2, POSTN, TAGLN and COL1A1) in n=3/4 primary NOF lines treated with TGFβ (4ng/mL), IL1β (1ng/mL)+TNF𝛼 (1ng/mL), monocyte conditioned media (CM) or LPS-activated monocyte conditioned media for 72 hours. Results show mean ± SD of n≥3 independent experiments, colours of points correspond to primary NOF line. One-way ANOVA with Bonferroni correction. *p < 0.05; **p < 0.01; ***p < 0.001. ****p < 0.0001.

**
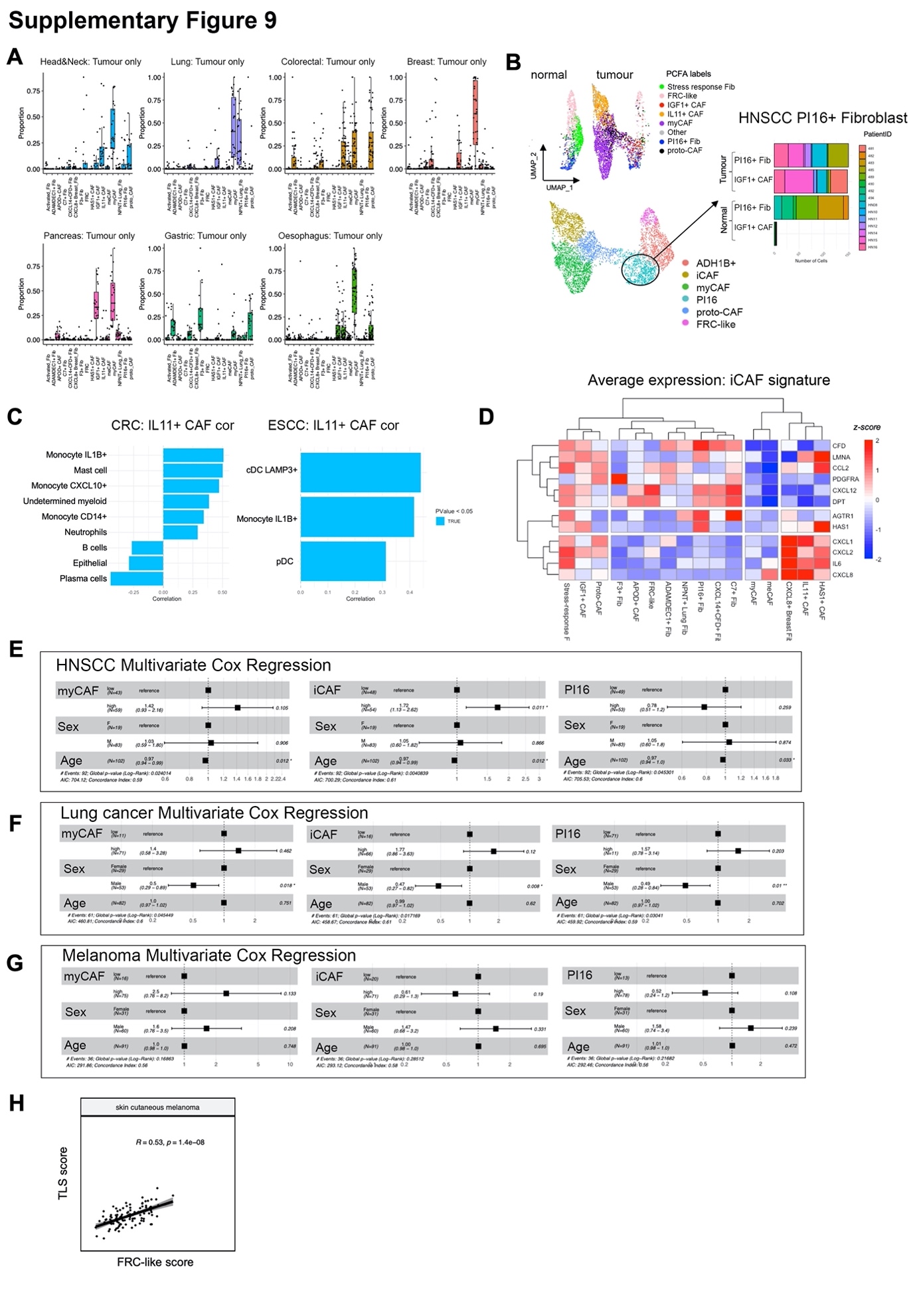
Supplementary Figure 9. Pan-Cancer Fibroblast Atlas (PCFA; related to Figure 5 & 6) and immunotherapy response (relating to Figure 7).**

A) Abundance of PCFA clusters in tumour sample only shown for each cancer type. Proportion of each cluster determined per sample. B) Transfer of labels from PCFA back to HNSCC UMAP (Figure 2) with accompanying bar plot showing the number of IGF1+ CAF or PI16+ fibroblasts in the original PI16 cluster (split by tumour or normal). IGF1+ CAF was specific to tumour samples, while PI16+ universal fibroblasts could be found in both. This supports previous analysis in Sup Fig 3, that in tumours, PI16 fibroblasts likely show early activation. C) Sample level scRNA-Seq correlations of IL11+ CAF with myeloid cell subsets in CRC and ESCC. Myeloid cell phenotypes were identified in CRC and ESCC datasets using label transfer – with HNSCC annotated myeloid cells acting as reference cells. Only significant correlations (spearman; p<0.05) are shown. D) Heatmap of iCAF signature [5] average expression in PCFA clusters. E) Forest plot for multivariate cox regression model using fibroblast (myCAF, IL11+ (i)CAF, PI16+ fibroblast) ssGSEA score (high or low), patient sex and patient age. Anti-PD-1/PD-L1 treated HNSCC cohort (GSE159067; n=102). Hazard ratio estimates along with confidence intervals (95%) and p-values are plotted for each variable. F) Forest plot for multivariate cox regression model using fibroblast (myCAF, IL11+ (i)CAF, PI16+ fibroblast) ssGSEA score (high or low), patient sex and patient age. Anti-PD-1/PD-L1 treated NSCLC cohort (GSE161537; n=82). Hazard ratio estimates along with confidence intervals (95%) and p-values are plotted for each variable. G) Forest plot for multivariate cox regression model using fibroblast (myCAF, IL11+ (i)CAF, PI16+ fibroblast) ssGSEA score (high or low), patient sex and patient age. Anti-CTLA-4 + anti-PD-1 or anti-PD-1 treated melanoma cohort (PRJEB23709; n=91). Hazard ratio estimates along with confidence intervals (95%) and p-values are plotted for each variable. H) TLS [6] and FRC-specific gene signature correlation in skin cutaneous melanoma. ssGSEA run using batch effects normalized mRNA data from the Pan-Cancer Atlas Hub (UCSCXena). Spearman correlation coefficient and p-value displayed. *p < 0.05; **p < 0.01; ***p < 0.001. ****p < 0.0001.

# **2. MATERIALS AND METHODS**

# **Primary fibroblast culture and *in vitro* experiments**

# Tissue disaggregation for cell culture

Upon receipt samples were washed once in Dulbecco’s modified eagle medium (Sigma #D5671) containing 10% Foetal Calf Serum, 1% Penicillin/streptomycin, 1% L-Glutamine, 1% Amphotericin, 1% Sodium pyruvate, and 12.5mM HEPES. The sample was chopped using a scalpel into 1-2mm size pieces prior to enzymatic digestion. All enzyme concentrations are final concentration. Enzymatic digestion was performed with collagenase-P (3 units.ml^-1^; Sigma #11213857001) and DNASE-1 (16 units.mL^-1^; Sigma #DN25) in cDMEM. The solution was sterile filtered using a 0.22µm syringe filter and the sample material was suspended in up to 5mL of cDMEM/enzyme solution. The sample was then sealed and placed in a benchtop shaker/incubator at 37^O^C and 150rpm. The samples were returned to the incubator (37^O^C / 150rpm) for up to a maximum of 45 minutes (or until digestion is complete) with trituration performed using a 5mL graduated pipette every 15 minutes. Any remaining undigested pieces were allowed to settle at the bottom of the tube; the supernatant was then transferred to a fresh sterile tube. Any remaining tissue was set aside. Complete DMEM, up to 10 mL, was added to each fraction and both cell suspensions were pelleted at 350rcf for 5 minutes. Supernatant was removed and RBC lysis buffer (Biolegend #420301) used to remove erythrocytes for 10 minutes at 4^o^C. The samples were then washed in PBS and suspended in residual volume, counted (haemocytometer), and viability assessed by Trypan blue exclusion. Cells were then plated at high density in plastic tissue culture flasks, with adherent cells then expanded until confluence was reached in T175 flasks. To create frozen stocks, fibroblasts were cryopreserved using standard procedures in FCS + 10% DMSO.

# Primary fibroblast culture

Primary fibroblasts were routinely cultured at 5% CO_2_/37°C in Dulbecco’s modified Eagle medium (DMEM; Sigma) supplemented with 10% foetal bovine serum (FBS), 1% (2mM) L-Glutamine (Sigma), 1% penicillin/streptomycin (Sigma) and 1% sodium pyruvate (Gibco). When adherent cells reached full confluency, media was removed, monolayer washed with phosphate-buffered saline (PBS), then trypsin-EDTA (Sigma) was added to detach monolayer. Fibroblasts were split 1:4 into new flasks in fresh media. Fibroblasts used in experiments were <12 passage.

# *In vitro* cytokine stimulation of fibroblasts

Primary NOFs were isolated from the mucosa of Head and Neck patient tissue samples as previously described. NOF were cultured in DMEM (Sigma D7561) supplemented with 10% FBS, 1% Penicillin/Streptomycin (Sigma P4333), 2mM L-Glutamine (Sigma G7513), 1mM sodium pyruvate (Sigma S8636). Fibroblast treatments included: 4ng/ml TGF-β1 (2-3 days; synthesised in-house), 1ng/ml TNF-α (2-3 days; R&D Systems, 210-TA), 1ng/ml IL-1β (2-3 days; R&D Systems, 201-LB), 50-100ng/ml Lymphotoxin-α1β2 (2-7 days; R&D Systems, 8884-LY-025) and 1μM TGF-β RI Kinase Inhibitor IV (2-7 days; Merk, 616454) with reduced serum (1% FBS) media.

# Conditioned-media treatment of fibroblasts

For monocyte-conditioned media (CM), human peripheral blood mononuclear cells (PBMC’s) were isolated from human leukocyte blood cones using Ficoll Paque (Cytiva 171444003) as per the manufacturer’s guidelines. For monocyte CM, PBMC’s were collected and 21x10^6^ cells seeded in a 100mm petri dish in RPMI-1640 (Sigma R8758) with 10% FBS for 2 hours. The adherent monocytes were subsequently washed with PBS to remove non adherent cells and incubated +/-1mg/ml LPS (Sigma L5293) in 5% FBS, for 2 hours. Media was removed and the cells were washed with PBS. RPMI (no FBS) was added for collection of the CM after 24 hours. NOF were cultured with CM for 72 hours, with comparative TNF-a/IL1-β treatment for the same time.

# qPCR, RNA extraction and cDNA synthesis

Fibroblasts were seeded per 6-well for qPCR analysis. RNA extraction was performed using a ReliaPrep RNA Miniprep Kit (Promega Z6012) following the standard protocol and quantified by NanoDrop (ThermoFisherScientific). mRNA was retro-transcribed using RevertAid First-Strand cDNA Synthesis Kit (Thermo Scientific K1691) following the manufacturer’s instructions. qPCR was performed with SYBR green reagent (Life Technologies). Analysis of relative gene expression was performed using ΔΔCT by comparison of the gene of interest CT value to housekeeping genes RPL41 CT value. Data were presented using Prism (GraphPad, v10). In conditions in which genes of interest were undetermined, Ct values were set to 40 and this was clearly indicated on the graphs.

# Primer sequences and concentrations:

ACTA2, 0.4µM, F:GACAATGGCTCTGGGCTCTGTAA/R:ATGCCATGTTCTATCGGGTACTT,

COL1A1,0.2µM,F:ACGAAGACATCCCACCAATCACCT/R:AGATCACGTCATCGCACAACACCT,

TAGLN,0.4µM,F:GGACCCTGATGGCTTTGGG/R:TATGCTCCTGCGCTTTCTTCATA,

POSTN,0.2µM,F:AAGGCTGCCCAGCAGTTTTG/R:CGATGCCCAGAGTGCCATAA,

MME,0.8µM,F:AGAAGTCGTTTTGAAAGATGTCCT/R:GCTTTTGCTTTCTGCACTGCT,

MMP3,0.4µM,F:TCCCTCAGGAAGCTTGAACCTGAA/R:AAACCTAGGGTGTGGATGCCTCTT,

IL6,0.2µM,F:AAATTCGGTACATCCTCGACGGCA/R:AGTGCCTCTTTGCTGCTTTCACAC,

IL11,0.8µM,F:CAGCACACCTGGGAGCTGTA/R:GACCACAACCTGGATTCCCT,

RPL41,0.4µM,F: CCATGAGAGCCAAGTGGAGGAA /R:TGGACCTCTGCCTCATCTTTC

CCL19,0.4µM,F:TGCCTGCTGTAGTGTTCACC/R:GCAGTCTCTGGATGATGCGT

CCL21,0.4µM,F:ATCCCAGCTATCCTGTTCTTGC/R:CAGAGCTCCTTTGGGTCTGC

RBP5,0.4µM,F:CCATAGTAACCTGGGAGGAGGA/R:AAGACCTGCTCGCACACTGCAT

SPIB,0.4µM,F:GAGGCTGCACAGCTCGAC/R: CGCCATCTGGGTACAGACAG

# **scRNA-Seq**

# scRNA-Seq sample processing

The finalised methodology for preparing HNSCC samples for the 10X Chromium pipeline was a two-stage digestion protocol designed to improve capture rates of stromal cells relative to immune cell subsets. The first stage of the enzymatic digestion was performed using Liberase^TM^ (Sigma #5401020001) at 100µg.mL^-1^ and DNASE-1 (Sigma #DN25) at 16 units.mL^-1^ in cDMEM. The solution was sterile filtered using a 0.22µm syringe filter and the sample material was suspended in up to 5mL of cDMEM/Liberase solution. This was allowed to digest for fifteen minutes (37^o^C/150rpm) and then supernatant removed (liberase fraction). For the second stage cDMEM containing collagenase-P (Sigma #11213857001) at 3 units.ml^-1^, liberase at 100µg.mL^-1^, dispase (Sigma #D4693) at 0.5 units.mL^-1^, elastase (Sigma #E1250) at 400µg.mL^-1^, trypsin (Sigma #T4799) at a final concentration of 0.25%, and DNASE-1 (16 units.mL^-1^) was added to the remaining material through a 0.22µm sterile syringe filter. This second digest will be referred to as Col+ going forward. The Col+ digest was returned to the incubator (37^o^C/150rpm) for up to a maximum of 45 minutes (or until digestion is complete), the supernatant was then transferred to a fresh sterile tube. Complete DMEM, up to 10 mL, was added to each fraction and both cell suspensions were pelleted at 350rcf for 5 minutes. Supernatant was removed and RBC lysis buffer (Biolegend #420301) used to remove erythrocytes for 10 minutes at 4^o^C. Each cell pellet was suspended in PBS containing 2% BSA-Fraction V (Scientific Lab Supplies #10735108001) and passed through a pre-wetted 40µm filter. Both samples were then counted, and viability assessed by Trypan blue exclusion. A final visual check of sample quality was also performed to ensure there were no large clumps of cells nor debris from the digestion. Finally, the two fractions were used to make a 100µL suspension of 100,000 cells of which 10,000 were from the liberase fraction and 90,000 from the Col+ fraction (1:9), and 2% BSA in PBS was used as the diluent. This cell suspension was then run immediately on the Chromium Controller.

# Sequence alignment and annotation

HPV reference sequences were downloaded from PaVE: The Papillomavirus Episteme (https://pave.niaid.nih.gov). The HPV-16 reference sequence (NC_001526) was used in the first instance and in cases requiring further identification of the HPV subtype references including HPV-33 (OQ_672679) and HPV-18 (NC_001357) were also created. In all cases the individual exons of HPV were identified in the FASTA and .GTF files to allow identification during alignment.

# Normalisation and integration of scRNA-Seq data

SCTransform (using variance-stabilizing transformation) was used to normalise each patient sample separately, before then integrating the dataset using Seurat’s reciprocal PCA (RPCA) integration workflow – selected due to the more conservative integrative approach and large cell number. scRNA-Seq integration was performed using IRIDIS High Performance Computing Facility (University of Southampton). SelectIntegration features, PrepSCTIntegration, FindIntegrationAnchors and IntegrateData were run specifying “SCT” normalisation method and using default parameters as outlined (<https://satijalab.org/seurat/articles/integration_rpca.html>).

# Doublet detection

We manually detected and subsequently removed doublets, carried out by incrementally increasing clustering resolutions and identifying DEGs (via FindMarkers). In addition to this, for fibroblast analysis, the assay was changed to “RNA” in the integrated cells, new variable features were found, followed by PCA and clustering in which distinct cell clusters expressing contaminating cell type markers were removed. Fibroblasts were then re-clustered using the original “integrated” assay (recalculating PCA, UMAP and clustering).

# HNSCC inter-dataset integration

We obtained scRNA-Seq data from GSE164690 [7]. The data was processed as outlined above, with 10% used as the mitochondrial gene percentage threshold. We omitted peripheral blood specimens and 1 HNSCC sample from the larynx to leave CD45/*PTPRC*+ and CD45/*PTPRC*- samples from oropharynx and oral cavity tumours. We initially integrated the datasets via splitting per patient sample for SCTransform and using Seurat’s RPCA integration again – generating an integrated HNSCC dataset of all cells (159,826 cells). However, for finer analysis of individual cell types we altered the method. Due to GSE164690 dataset consisting of CD45-/+ fractions sorted via fluorescence-activated cell sorting prior to scRNA-Seq, we separated our in-house dataset into CD45+ and CD45- objects based on the annotated integrated UMAP. Seurat’s RPCA integration was carried out separately (as outlined previously) to generate two integrated HNSCC objects, one containing immune cells only (CD45+) and another for non-immune cells (CD45-). Subsetting of clusters of interest was performed in which PCA was recalculated in subset of cells followed by UMAP, clustering and manual annotation; with contaminating clusters removed.

# **Pan-Cancer Fibroblast Atlas (PCFA)**

# Datasets

We selected datasets for the PCFA that contained both normal and (primary) tumour samples and were generated using the 10x Chromium platform. The datasets are as follows. Lung datasets (Kim_Lee_2020, He_Fan_2021, Lambrechts_Thienpont_2018_6149v2) were retrieved from the integrated NSCLC transcriptome atlas (<https://cellxgene.cziscience.com/collections/edb893ee-4066-4128-9aec-5eb2b03f8287>) [8]. The following datasets were downloaded from the Gene Expression Omnibus: Breast (GSE161529), Gastric (GSE150290), Oesophageal (GSE160269) and Colorectal (GSE178341). The pancreatic cancer dataset was retrieved from the Genome Sequence Archive under project PRJCA001063.

# Workflow

Each dataset was initially processed separately, in which the 10x feature matrices were read in along with patient metadata and Seurat objects were object generated (min.cells = 3, min.features =200). QC involved nfeatures ≤ 6000 and mitochondrial gene percentage ≤ 20%. Genes were converted to official gene symbols if required. NormalizeData, FindVariableFeatures, ScaleData, RunPCA, FindNeighbors, FindClusters, RunUMAP were then applied to cluster cells. Immune cells (e.g., PTPRC) and epithelial cells (e.g., *EPCAM*) were initially removed based on marker gene expression – leaving mesenchymal cells (fibroblasts, endothelial cells, mural cells, Schwann cells, mesothelial cells). Mesenchymal cell objects were created for all datasets then loaded into the same environment. Datasets were merged and a common gene space was determined (13,734 genes). We then leveraged Seurat v5 (v5.0.1) sketch-based integration (<https://satijalab.org/seurat/articles/parsebio_sketch_integration>) to perform integration using 2500 cells per dataset. HarmonyIntegration method [9] was chosen in which the dataset and Chromium kit version were harmonised (theta = 1). ProjectIntegration and ProjectData (1:40 dims) were then used to integrate the full datasets. Clusters expressing *LUM* and negative for other cell type markers (*PTPRC, EPCAM, RGS5, VWF, S100B, MSLN*) were subset and the process outlined above was repeated on the LUM+ cells to generate the PCFA.

# Pseudobulk

Marker genes between PCFA were found using FindAllMarkers as outlined previously. Pseudobulk differential expression analysis was carried out using AggregateExpression to generate pseudobulk samples grouped by cluster, dataset, and patient sample. FindMarkers was used with ‘DESeq2’ method.

# Label Transfer

We used a Label transfer approach to identify myeloid cell phenotypes in CRC and Oesophageal scRNA-Seq datasets. The integrated HNSCC myeloid cells were used as the reference dataset. CRC and Oesophageal datasets were first normalised with SCTransform, anchors were identified using FindTransferAnchors (reduction = pcaproject, projecting the PCA from the reference onto the query) and phenotype predictions made using TransferData (1:30 dims). Predicted cell type labels with prediction.score.max ≥ 0.5 were annotated. The relative immune cell abundance of each myeloid cell phenotype was then calculated per tumour sample and correlated with relative fibroblast abundance (using the PCFA) to verify myeloid and fibroblast associations.

# **Spatial transcriptomics**

Count matrices for each of 10 samples were imported into Seurat and normalized using the SCTransform. The samples were integrated using Seurat v3's CCA anchor finding method (FindIntegrationAnchors and IntegrateData). The 3000 variable features selected for integration were then used for principal component analysis (PCA), followed by the FindNeighbors and FindClusters functions for (shared) nearest-neighbor graph construction and cluster determination, respectively. Resulting clusters were examined, with poor-quality clusters being removed. Spatial feature expression plots were created using the SpatialFeaturePlot function in Seurat.

Spot Deconvolution

To deconvolve Visium spots into encompassing cell types, Robust Cell Type Decomposition (RCTD) was utilized with spacexr 2.2.1 [10] in R. This analysis used the annotated scRNA-Seq HNSCC reference dataset. RCTD was executed on each individual patient sample with default parameters, and the doublet mode set to ‘full’. The resulting normalized weights for each cell type were then obtained. For subsequent correlation analysis we implemented a minimum abundance (normalized weight) threshold of 0.05 (assuming each spot could contain a maximum of 20 cells). In addition, SpatialDWLS [11] was also used to validate spot deconvolution using the Giotto package (v3.3.1). Briefly, Seurat objects were converted to Giotto objects using seuratToGiotto function, then guidelines were followed from the Giotto website. The annotated scRNASeq HNSCC dataset was used to create a reference expression matrix (with mitochondrial and ribosomal genes removed), which was normalised and the top 100 genes (using findMarkers_one_vs_all, method = ‘scran’) for each cell type were selected for DWLS matrix construction. runDWLSDeconv was then ran on each giotto object separately with n_cell parameter set to 20. When cell type correlations were performed separately for each patient, the weighted Fisher’s method was used to combine p values to find significant associations.

# Ligand Receptor and NicheNet analysis:

We first obtained the curated ligand-receptor interaction network from NicheNet [lr_network_human_21122021 (lr_network)] [12]. We identified spatially differentially expressed (DE) ligands and receptors by findings DEGs between fibroblast-containing spots (RCTD normalised weight ≥ 0.05) compared to all other spots across all patient samples. DEGs (logfc.threshold = 1 and minimum expression in 25% of spots) were then filtered using the lr_network ligands and receptors. For each fibroblast subset, expressed receptors were identified using get_expressed_genes (minimum expression detected in ≥25% cells) and filtering for receptors in the lr_network. We then filtered the lr_network to retain spatially DE ligands (niche ligands) that fibroblasts expressed the corresponding receptor to. Finally, we identified unique niche ligands (not spatially DE in other fibroblast niches). Sender cells were identified by scRNA-Seq and spatial transcriptomic cell type correlations. Within each ‘Sender’ cell type: ligands expressed by ≥ 10% of cells with mean normalised expression ≥0.1 were selected. This formulated niche-specific ligand-receptor pairs.

NicheNet was used to perform ligand activity analysis – identifying ligands ranked based on the area under the precision-recall curve (AUPR) – as outlined in the vignette. Sender cell types were selected based on prior spatial analysis and included the fibroblast subset of interest. DEGs between fibroblasts were determined (min.pct = 0.25, logfc.threshold = 1) and the top 100 genes (avg_log2FC ≥1.5; ordered by descending avg_log2FC) were selected as the gene sets of interest. predict_ligand_activities was then used to determine ligand activities of niche-specific ligand-receptor pairs which were then sorted by descending aupr_corrected values.

# **Generation of gene signatures for fibroblasts**

Fibroblast-specific gene signatures were generated as follows (Schematic 1) in a similar manner to previous studies [13–15]. 1) Identification of DEGs between HNSCC fibroblast clusters. 2) Identification of DEGs between fibroblast cluster and all other HNSCC cell types (removing genes expressed in >10% of other cell types). 3) Filter step 1 DEGs by step 2 DEGs. 4) Filter to retain genes expressed by ≥ 50% of cells within cluster. The top 20 genes were then selected, and co-expression (spearman’s rho) was assessed using the TCGA HNSC bulk RNA-Seq data. Only genes that significantly positively correlated were retained to formulate the signature (Supplementary Table 8).

Schematic 1. Selection of genes used for ssGSEA of bulk RNA-Seq data.

# **Multiplex immunofluorescence using PhenoCycler-Fusion (CODEX)**

5 µm FFPE tissue sections were used to perform PhenoCycler-Fusion (CODEX) high order mIF assays according to manufacturers’ recommendation (Akoya Biosciences, MA, USA). Briefly, slides were baked at 60°C for 1 h, followed by a series of deparaffinization and rehydration steps. Target retrieval was done in a pressure cooker for 20 min with Tris/EDTA buffer, pH 9 (Dako). Slides were cooled at room temperature, rinsed, and stored in Hydration Buffer at 4°C until staining. For the staining, an antibody cocktail was applied with preoptimized dilutions of each antibody and incubated at room temperature for 3 h. Slides were washed with staining buffer, followed by three-step fixation with 1.6% PFA, methanol, and fixative reagent. For the imaging, a flow cell (Akoya Biosciences) was assembled and two blank cycles without reporters were used as the first and last cycles for the subtraction of autofluorescence background on the PhenoCycler-Fusion imaging system (Akoya Biosciences). The Imager acquires DAPI and three channels (Cy3, Cy5, Cy7) at a time and generates a final QPTIFF file containing a composite image of all markers. Antibodies and reporters used in PhenoCycler-Fusion assays were purchased from Akoya Biosciences.

| **Antibody** | **Dilution** | **Clone** | **Catalog #** | **Barcode/Reporter** |
| --- | --- | --- | --- | --- |
| CD34 | 1:100 | QBEND/10 | 4250057 | BX025/RX025-ATTO550 |
| CD4 | 1:200 | EPR6855 | 4550112 | BX003/RX003-Cy5 |
| CD20 | 1:200 | L26 | 4450018 | BX007/RX007-AF750 |
| CD14 | 1:500 | EPR3653 | 4450047 | BX037/RX037-ATTO550 |
| CD68 | 1:100 | KP1 | 4550113 | BX015/RX015-Cy5 |
| CD31 | 1:100 | EP3095 | 4450017 | BX001/RX001-AF750 |
| CD45 | 1:300 | D9M81 | 4550121 | BX021/RX021-Cy5 |
| SMA | 1:200 | 1A4 | 4450049 | BX013/RX013-AF750 |
| CD3e | 1:200 | EP449E | 4550119 | BX045/RX045-Cy5 |
| Pan-Cytokeratin | 1:500 | AE1/AE3 | 4450020 | BX019/RX019-AF750 |
| Podoplanin | 1:200 | NC-08 | 4250004 | BX023/RX023-ATTO550 |
| FoxP3 | 1:100 | 236A/E7 | 4550071 | BX031/RX031-AF647 |
| CD163 | 1:100 | D6U1J | Custom | BX016/RX016-AF647 |
| CD21 | 1:300 | EP3093 | 4450027 | BX032/RX032-ATTO550 |
| MPO | 1:200 | E1E7I | 4250083 | BX098/RX098-ATTO550 |

**References**

1. Hynes RO, Naba A. Overview of the matrisome--an inventory of extracellular matrix constituents and functions. Cold Spring Harb Perspect Biol. 2012;4:a004903.

2. Coppola D, Nebozhyn M, Khalil F, Dai H, Yeatman T, Loboda A, et al. Unique ectopic lymph node-like structures present in human primary colorectal carcinoma are identified by immune gene array profiling. Am J Pathol. 2011;179:37–45.

3. Hennequin A, Derangère V, Boidot R, Apetoh L, Vincent J, Orry D, et al. Tumor infiltration by Tbet+ effector T cells and CD20+ B cells is associated with survival in gastric cancer patients. Oncoimmunology. 2016;5:e1054598.

4. Gu-Trantien C, Loi S, Garaud S, Equeter C, Libin M, de Wind A, et al. CD4+ follicular helper T cell infiltration predicts breast cancer survival. J Clin Invest. 2013;123:2873–92.

5. Elyada E, Bolisetty M, Laise P, Flynn WF, Courtois ET, Burkhart RA, et al. Cross-Species Single-Cell Analysis of Pancreatic Ductal Adenocarcinoma Reveals Antigen-Presenting Cancer-Associated Fibroblasts. Cancer Discov. 2019;9:1102–23.

6. Cabrita R, Lauss M, Sanna A, Donia M, Skaarup Larsen M, Mitra S, et al. Tertiary lymphoid structures improve immunotherapy and survival in melanoma. Nature. 2020;577:561–5.

7. Kürten CHL, Kulkarni A, Cillo AR, Santos PM, Roble AK, Onkar S, et al. Investigating immune and non-immune cell interactions in head and neck tumors by single-cell RNA sequencing. Nat Commun. 2021;12:7338.

8. Salcher S, Sturm G, Horvath L, Untergasser G, Kuempers C, Fotakis G, et al. High-resolution single-cell atlas reveals diversity and plasticity of tissue-resident neutrophils in non-small cell lung cancer. Cancer Cell. 2022;40:1503-1520.e8.

9. Korsunsky I, Millard N, Fan J, Slowikowski K, Zhang F, Wei K, et al. Fast, sensitive and accurate integration of single-cell data with Harmony. Nat Methods. 2019;16:1289–96.

10. Cable DM, Murray E, Zou LS, Goeva A, Macosko EZ, Chen F, et al. Robust decomposition of cell type mixtures in spatial transcriptomics. Nat Biotechnol. 2022;40:517–26.

11. Dong R, Yuan G-C. SpatialDWLS: accurate deconvolution of spatial transcriptomic data. Genome Biol. 2021;22:145.

12. Browaeys R, Saelens W, Saeys Y. NicheNet: modeling intercellular communication by linking ligands to target genes. Nat Methods. 2020;17:159–62.

13. Kieffer Y, Hocine HR, Gentric G, Pelon F, Bernard C, Bourachot B, et al. Single-Cell Analysis Reveals Fibroblast Clusters Linked to Immunotherapy Resistance in Cancer. Cancer Discov. 2020;10:1330–51.

14. Dominguez CX, Müller S, Keerthivasan S, Koeppen H, Hung J, Gierke S, et al. Single-Cell RNA Sequencing Reveals Stromal Evolution into LRRC15+ Myofibroblasts as a Determinant of Patient Response to Cancer Immunotherapy. Cancer Discov. 2020;10:232–53.

15. Buechler MB, Pradhan RN, Krishnamurty AT, Cox C, Calviello AK, Wang AW, et al. Cross-tissue organization of the fibroblast lineage. Nature. 2021;593:575–9.
